# Methylome analysis of extreme chemoresponsive patients identifies novel markers of platinum sensitivity in high-grade serous ovarian cancer

**DOI:** 10.1186/s12916-017-0870-0

**Published:** 2017-06-23

**Authors:** Tushar Tomar, Nicolette G. Alkema, Leroy Schreuder, Gert Jan Meersma, Tim de Meyer, Wim van Criekinge, Harry G. Klip, Heidi Fiegl, Els van Nieuwenhuysen, Ignace Vergote, Martin Widschwendter, Ed Schuuring, Ate G. J. van der Zee, Steven de Jong, G. Bea A. Wisman

**Affiliations:** 1 Department of Gynecologic Oncology, Cancer Research Center Groningen, University of Groningen, University Medical Center Groningen, PO Box 30001, 9700 RB Groningen, The Netherlands; 2 Department of Mathematical Modelling, Statistics and Bioinformatics, Ghent University, Ghent, Belgium; 3 Department of Obstetrics and Gynecology, Medical University of Innsbruck, Innsbruck, Austria; 4 Division of Gynecological Oncology, Department of Obstetrics and Gynecology, Leuven Cancer Institute, University Hospitals Leuven, Leuven, Belgium; 5 Department of Women’s Cancer, UCL Elizabeth Garrett Anderson Institute for Women’s Health, University College London, London, UK; 6 Department of Medical Biology and Pathology, Cancer Research Center Groningen, University of Groningen, University Medical Center Groningen, Groningen, The Netherlands; 7 Department of Medical Oncology, Cancer Research Center Groningen, University of Groningen, University Medical Center Groningen, PO Box 30001, 9700 RB Groningen, The Netherlands

**Keywords:** DNA methylation, Integrated methylome analysis, Ovarian cancer, Platinum-based chemotherapy, Extreme chemoresponders

## Abstract

**Background:**

Despite an early response to platinum-based chemotherapy in advanced stage high-grade serous ovarian cancer (HGSOC), the majority of patients will relapse with drug-resistant disease. Aberrant epigenetic alterations like DNA methylation are common in HGSOC. Differences in DNA methylation are associated with chemoresponse in these patients. The objective of this study was to identify and validate novel epigenetic markers of chemoresponse using genome-wide analysis of DNA methylation in extreme chemoresponsive HGSOC patients.

**Methods:**

Genome-wide next-generation sequencing was performed on methylation-enriched tumor DNA of two HGSOC patient groups with residual disease, extreme responders (≥18 months progression-free survival (PFS), *n* = 8) and non-responders (≤6 months PFS, *n* = 10) to platinum-based chemotherapy. DNA methylation and expression data of the same patients were integrated to create a gene list. Genes were validated on an independent cohort of extreme responders (*n* = 21) and non-responders (*n* = 31) using pyrosequencing and qRT-PCR. In silico validation was performed using publicly available DNA methylation (*n* = 91) and expression (*n* = 208) datasets of unselected advanced stage HGSOC patients. Functional validation of *FZD10* on chemosensitivity was carried out in ovarian cancer cell lines using siRNA-mediated silencing.

**Results:**

Integrated genome-wide methylome and expression analysis identified 45 significantly differentially methylated and expressed genes between two chemoresponse groups. Four genes *FZD10*, *FAM83A*, *MYO18B*, and *MKX* were successfully validated in an external set of extreme chemoresponsive HGSOC patients. High *FZD10* and *MKX* methylation were related with extreme responders and high *FAM83A* and *MYO18B* methylation with non-responders. In publicly available advanced stage HGSOC datasets, *FZD10* and *MKX* methylation levels were associated with PFS. High *FZD10* methylation was strongly associated with improved PFS in univariate analysis (hazard ratio (HR) = 0.43; 95% CI, 0.27–0.71; *P* = 0.001) and multivariate analysis (HR = 0.39; 95% CI, 0.23–0.65; *P* = 0.003). Consistently, low *FZD10* expression was associated with improved PFS (HR = 1.36; 95% CI, 0.99–1.88; *P* = 0.058). *FZD10* silencing caused significant sensitization towards cisplatin treatment in survival assays and apoptosis assays.

**Conclusions:**

By applying genome-wide integrated methylome analysis on extreme chemoresponsive HGSOC patients, we identified novel clinically relevant, epigenetically-regulated markers of platinum-sensitivity in HGSOC patients. The clinical potential of these markers in predictive and therapeutic approaches has to be further validated in prospective studies.

**Electronic supplementary material:**

The online version of this article (doi:10.1186/s12916-017-0870-0) contains supplementary material, which is available to authorized users.

## Background

Epithelial ovarian cancer is the most lethal gynecologic malignancy [1]. High-grade serous ovarian cancer (HGSOC), the most abundant histological subtype of ovarian cancer, is generally diagnosed at an advanced stage. Standard care of advanced stage patients includes debulking surgery in combination with platinum-based chemotherapy in an adjuvant or neoadjuvant setting. Unlike many other epithelial cancers, HGSOC is initially hypersensitive to platinum chemotherapy. However, up to 75% of responding patients relapse with platinum-resistant disease, resulting in a 5-year survival rate of below 40% [2, 3]. Furthermore, if a relapse occurs within 6 months after initial treatment (progression-free survival (PFS) ≤ 6 months), the patient is regarded as ‘platinum resistant’ [4, 5]. Based on clinicopathological parameters, it is difficult to identify patients who will respond to platinum chemotherapy. As a surrogate indicator for platinum sensitivity, robust biomarkers associated with very short PFS might help identify relapse-prone patients. Instead of undergoing platinum-based chemotherapy, they could be selected for other novel treatment regimes.

HGSOC differs from other malignancies regarding the prevalence of somatic gene mutations. Except for the frequent inactivating mutation of tumor suppressor *TP53* (96%) and mutations of the *BRCA1/2* (20%) from the DNA damage repair pathway, mutations in other genes are rare [6, 7]. However, progression of HGSOC involves abundant epigenetic alterations, mainly DNA methylation redistribution, which is characterized by global genomic hypomethylation and localized hypermethylation [6, 8]. Besides the relative stability of DNA methylation, hypermethylation is functionally related to gene expression and can be easily analyzed in body fluids [9, 10]. Therefore, DNA methylation can be used as a clinical biomarker.

To date, several studies have been conducted to find robust DNA methylation biomarkers for ovarian cancer. Many specific hypermethylated genes have been reported as potentially useful for diagnosis, prognosis, and/or sometimes for chemoresponse [11–13]. However, most of these studies included all histological subtypes of ovarian cancer and were predominantly based on a single candidate gene approach. Only a few studies have described the identification of platinum chemoresponse methylation markers in HGSOC [14–16]. These studies were based on customized or commercially available methylation array-based platforms, and were limited by the number of CpG sites or to genes of specific pathways.

The aim of the present study was to identify putative methylation markers for chemoresponse in HGSOC. We took an unbiased genome-wide approach and determined the methylation status of PFS-based extreme chemoresponder and non-responder HGSOC patients by performing enrichment of methylated DNA using the methyl-CpG binding domain of MeCP2 protein followed by next generation-sequencing (MethylCap-seq). The differentially methylated profile between extreme responders and non-responders was integrated with microarray expression data to identify putative methylation markers for chemoresponse in HGSOC. In addition, our findings were validated in an independent patient cohort of extreme responders and non-responders, which resulted in *FZD10*, *FAM83A*, *MYO18B*, and *MKX* as candidate chemoresponse markers. In silico validation of candidate genes was performed using publicly available DNA methylation and expression datasets of unselected advanced stage HGSOC patients to assess their predictive value. Finally, we functionally validated *FZD10* involvement in platinum sensitivity using in vitro models.

## Methods

### Patient population involved

This retrospective study was conducted in agreement with the Reporting Recommendations for Tumor Marker Prognostic Studies (REMARK) criteria for reporting tumor biomarker prognostic studies [17]. A checklist for the criteria is provided (Additional file 1).

#### Set 1 (frozen tissue, University Medical Center Groningen (UMCG))

The discovery set consisted of prospectively collected chemo-naïve frozen tumor tissue of 18 patients with advanced-stage HGSOC operated by a gynecologic oncologist from the UMCG (Groningen, the Netherlands) in the period 1990–2008. All patients were staged according to the International Federation of Gynecology and Obstetrics (FIGO) guidelines. Standard treatment included debulking surgery followed by adjuvant chemotherapy consisting of platinum-based treatment regimens. After chemotherapy, patients were followed for up to 10 years with gradually increasing intervals. All the clinicopathological and follow-up data have been registered in an anonymous, password-protected database, in compliance with Dutch law. All patients gave informed consent. The responder group consisted of patients with advanced stage HGSOC, residual disease after primary surgery (> 2 cm), treated with adjuvant platinum-based chemotherapy and a PFS of more than 18 months. The non-responder group consisted of patients with advanced stage HGSOC, residual disease after primary surgery (> 2 cm), treated with adjuvant platinum-based chemotherapy and a PFS of less than 6 months. We have *p53* and *BRCA1/2* status information for 17 (8 responders and 9 non-responders) out of 18 discovery dataset patients. In this cohort, 16 were *p53* mutated, except for one non-responder, and only two responders showed *BRCA2* germline mutation. Detailed clinicopathological features are described in Additional file 2: Table S1.

#### Set 2 (mRNA dataset, UMCG)

This previously published gene expression data included 157 consecutive advanced-stage HGSOC patient samples from UMCG profiled using two-color oligonucleotide microarrays (35,000 Operon v3.0 probes), manufactured by The Netherlands Cancer Institute (Amsterdam, the Netherlands, https://www.nki.nl/topmenu/genomics-core-facility/, GSE 13876) as described by Crijns et al. [18]. For expression data integration, we used data from 11 patients (6 responders and 5 non-responders) who were also in the discovery set for MethylCap-Seq. Detailed clinicopathological features are described in Additional file 2: Table S1.

#### Set 3 (frozen tissue; UMCG + Innsbruck + Leuven)

The external validation cohort consisted of HGSOC patient tumors from 21 responders and 31 non-responders obtained from the UMCG, the Medical University of Innsbruck (Austria), and University Hospital Leuven (Belgium). All patients were selected based on the same inclusion criteria as the discovery set (Set 1). Detailed clinicopathological features are described in Additional file 2: Table S1.

#### Sets 4, 5, and 6 (Publicly available external cohort data)

For in silico validation of our findings, we used publicly available methylation and expression datasets from HGSOC patients. For methylation Set 4, Infinium 450K methylation array data of AOCS study group (http://www.aocstudy.org.) was extracted from the NCBI GEO portal using GEO accession no. GSE65820 [19] (http://www.ncbi.nlm.nih.gov/geo/query/acc.cgi?acc=GSE65820) and normalized using a beta-mixture quantile normalization as described previously [20]. The clinical data of patients was downloaded from the ICGC data portal (https://dcc.icgc.org/). Methylation probes for *FZD10* (cg23054883), *FAM83A* (cg24833277), *MYO18B* (cg24035545), and *MKX* (cg14947429), which are related to same marker regions as identified with MethylCap-seq (shown in Fig. 2a for *FZD10*), have been used for univariate Mantel–Cox log-rank survival analysis to generate Fig. 4a, b and Additional file 3: Figure S4–S6. For methylation Set 5, Infinium 27K methylation array data of TCGA study group along with associated clinical information was extracted from Genomic Data Commons portal (https://gdc.cancer.gov/). Data was beta-mixture quantile normalized and the *FZD10* methylation probe (cg23054883) was used for univariate Mantel–Cox log-rank survival analysis to generate Additional file 3: Figure S3.

Gene expression data from tumors of Set 6, the curated ovarian cancer database (Gyorffy et al*.* database, http://kmplot.com) [21], with carefully curated clinical annotations, was extracted in November 2015. We restricted our analysis to primary, high-grade (3), advanced stage (3 and 4), serous ovarian tumors, residual disease after surgery or suboptimal debulking, and platinum-containing therapy with available PFS and overall survival (OS). For candidate genes, we used the expression data of *FZD10* (Probe ID: 219764_at), *FAM83A* (Probe ID: 239586_at), *MYO18B* (Probe ID: 1554579_a_at), and *MKX* (Probe ID: 239468_at) to perform univariate Mantel–Cox log-rank survival analysis and to generate Fig. 4c, d and Additional file 3: Figure S4–S6.

Detailed clinicopathological features are described in Additional file 2: Table S1.

### DNA extraction and bisulfite modification

Histologic slides of patients were reviewed to confirm the diagnosis by an experienced gynecologic pathologist. Representative frozen blocks of each patient tumor were retrieved for DNA extraction. Frozen sections of 10 µm thickness were cut with periodic 4 µm sections prior to hematoxylin and eosin staining to assess the vital tumor cell percentage. For some samples, slides were macro-dissected to obtain more than 85% neoplastic cells. DNA was isolated using standard salt-chloroform extraction and isopropanol precipitation. Precipitated DNA was re-suspended in Tris-EDTA buffer (10 mM Tris; 1 mM EDTA, pH 8.0). Genomic DNA was amplified with multiplex PCR according to the BIOMED-2 protocol to check the DNA’s structural integrity [22]. DNA quantity was measured using Quant-iT™ PicoGreen® dsDNA Assay kit according to the manufacturer’s protocol (Invitrogen, Carlsbad, CA, USA). For DNA isolation from cell lines, the same standard method was followed. Bisulfite conversion was performed using EZ DNA methylation^tm^ Kit (Zymo Research, Orange, CA, USA) according to the manufacturer’s protocol using 1 μg of DNA.

#### MethylCap-seq

MethylCap-seq was performed as described previously [23, 24]. Briefly, methylated DNA fragments were captured with methyl binding domains using the MethylCap kit according to manufacturer’s instructions (Diagenode, Liège, Belgium). The kit consists of the methyl binding domain of human MeCP2 as a C-terminal fusion with glutathione-S-transferase containing an N-terminal His6-tag. Before capturing, DNA samples (500 ng) were sheared to a size range of 300–1000 bps using a Bioruptor™ UCD-200 (Diagenode, Liège, Belgium) and fragments of approximately 300 bp were isolated. Captured DNA was paired-end-sequenced on the Illumina Genome Analyzer II platform according to the protocol (Illumina, San Diego, CA, USA). Results were mapped on the nucleotide sequence using Bowtie software [25], visualized using BioBix’ H2G2 browser (http://h2g2.ugent.be/biobix.html) and processed using the human reference genome (NCBI build 37). The paired-end fragments were unique and located within 400 bp of each other. The MethylCap-seq data have been deposited in the Gene Expression Omnibus under accession number GSE97128.

#### Bisulfite pyrosequencing

Based on the next-generation sequencing results of the discovery set (Set 1), all pyrosequencing primers were designed for the selected candidate differentially methylated regions (DMRs) of 45 genes using PyroMark Assay Design software (Qiagen, Hilden, Germany). Bisulfite pyrosequencing was performed as described previously [26]. Briefly, bisulfite treated DNA was amplified using PyroMark PCR kit (Qiagen). PCR reaction and cycling conditions were according to the kit manual. To generate the PCR product from bisulfite-converted DNA, we adopted the amplification protocol using a universal primer approach as described by Collela et al*.* [27]*.* The biotinylated PCR products were captured using 1 μL streptavidin-coated sepharose high-performance beads (GE Healthcare, Little Chalfont, UK). The immobilized products were washed with 70% alcohol, denatured with PyroMark denaturation solution (Qiagen) and washed with PyroMark wash buffer (Qiagen). The purified PCR product was then added to 25 μL PyroMark annealing buffer (Qiagen) containing 0.3 μM sequencing primers for specific genes (primer sequences are given in Additional file 4). Finally, pyrosequencing™ reactions were performed in a PyroMark Q24 MD System (Qiagen) according to the manufacturer’s instructions using the PyroGold Q24™ Reagent Kit (Qiagen). CpG site methylation quantification was performed using the methylation Software Pyro Q24 2.06 Version (Qiagen).

### Cell line culturing

A panel of human ovarian cancer cell lines, A2780, C30, Cp70, SKOV3, OVCAR3, IGROV1, PEA1, PEA2, PEO14, and PEO23, was used for in vitro validation and functional analysis. The source, media, and culture conditions for the cell lines are shown in Additional file 2: Table S2. All cells were grown at 37 °C in a humidified atmosphere with 5% CO_2_ and were detached with 0.05% trypsin in phosphate-buffered saline (PBS, 0.14 mM NaCl, 2.7 mM KCl, 6.4 mM Na_2_HPO_4_, 1.5 mM KH_2_PO_4_, pH 7.4). Authenticity of all cell lines was verified by DNA short tandem repeat analysis (Baseclear, Leiden, The Netherlands) and mycoplasma testing was performed using in-house developed PCR-based method with specific primers (Invitrogen, NY) against various mycoplasma species. For global demethylation, cells at 40–50% confluency were treated with demethylating agent 5-aza-2′-deoxycytidine (DAC) at a final concentration of 1 μM for 72 h. Due to the low stability of DAC at 37 °C, the medium was replenished with DAC every 24 h. After 72 h, cells were trypsinized and processed for RNA and DNA isolation.

### Total RNA isolation and quantitative reverse transcriptase PCR (qRT-PCR)

qRT-PCR was performed as described previously [26]. Total RNA was isolated from frozen tissue blocks and cell lines using the same procedure as described for DNA extraction. Total RNA was isolated using RNeasy mini kit (Qiagen) according to the manufacturer’s instructions. RNA was analyzed quantitatively using Nanodrop (Nanodrop Technologies, Rockland, DE), by using 1 μg of total RNA for cDNA synthesis by a RNase H+ reverse transcriptase using iScript cDNA synthesis kit (BioRad, Hercules, CA) according to the manufacturer’s instructions. qRT-PCR was performed in an ABI PRISM 7900HT Sequence Detector (Applied Biosystems, Foster City, CA) with the iTaq SYBR Green Supermix with Rox dye (Biorad). The reactions were analyzed with SDS software (Version 2.4, Applied Biosystems). The threshold cycles (Ct) were calculated and relative gene expression (∆Ct) was analyzed with GAPDH as the housekeeping gene (∆Ct = Ct_gene_ – Ct_GAPDH_) (primer sequences given in Additional file 4). The qRT-PCR primers used are available upon request. For the final analysis, data was imported to R to perform clustering and ggplot2 (http://ggplot2.org/) was used to create heat maps.

### siRNA mediated silencing for in vitro experiments

Cells (1–3 × 10^5^) were plated in a 6-well plate and grown overnight. FZD10 trisilencer-27 siRNAs (Origene Technologies, Rockville, MD) were used for transient knock-down using 20 nM of final concentration of siRNA (sequences given in Additional file 4). Scrambled and FZD10 targeted siRNAs were transfected using Oligofectamine (Invitrogen, NY) for 4 h with reduced growth factor serum-free opti-MEM media (Gibco, Life Technologies, CA). Subsequently, cell line-associated media (Additional file 2: Table S2) with 30% FCS were added to make a final FCS concentration of 10% for 48 h. Following 48 h after siRNA transfection, other functional assays (short- and long-term survival, migration, and apoptosis) were performed.

### Short- and long-term survival assays

Short-term cellular viability was measured with the micro-culture tetrazolium assay (MTT) as described previously [28]. Briefly, in a 96-well culture plate, approximately 7500 SKOV3 cells, 15,000 OVCAR3 cells, 10,000 PEA2 cells, and 12,000 C-30 cells, either control or siRNA transfected, were seeded in 200 μL culture medium with or without cisplatin treatment. After 96 h, 20 μL of 3-(4,5-dimethythiazol-2-yl)-2,5-diphenyltetrazolium bromide (MTT, Sigma-Aldrich, St. Louis, MO, 5 mg/mL in PBS) was added and formazan production was measured colorimetrically using a Biorad iMark microplate reader at 520 nm wavelength.

For long-term assay, depending on the concentration of cisplatin, cells were seeded in 96-well plates at approximately 2000 cells per well for SKOV3 and 4000 cells per well for OVCAR3. After 8–10 h, indicated doses of cisplatin were added and allowed to grow for a set number of days. Finally, cells were fixed and stained in staining buffer (methanol (50%), acetic acid (20%) and 0.01% Coomassie brilliant blue), washed with water and dried, after which the plates were scanned. For quantification, 200 μL of 10% acetic acid was added to each well and left on a shaker for 30–60 min. Plates were read using a Biorad iMark microplate reader at 520 nm wavelength.

### Wound healing assays

For the wound healing assays, cells were seeded in a 6-well plate at a density of 2 × 10^5^ cells/well and grown overnight until confluency. A wound was created by manually scraping the cell monolayer with a 10 μL pipette tip and the medium was aspirated to remove the detached cells. Cells were then incubated with medium supplemented only with 10% FCS, and wound closure was observed within 24 h. Images were acquired with a Leica camera mounted on an inverted microscope and were processed using Image J software (NIH, Bethesda, MD; http://rsb.info.nih.gov/ij/). The distance cells migrated was determined by measuring the wound area at different time points followed by its correction from the wound area at time 0 h.

### Western blot analysis

Various proteins in ovarian cancer cell lines were detected by the western blotting method as described previously [28]. Western blot membranes were probed overnight at 4 °C with primary antibodies (PARP, Cell Signaling Technology, Danvers, MA, #9532; Cleaved Caspase-3, Cell Signaling Technology, #9661). Afterwards, HRP-conjugated secondary antibodies (DAKO, Glostrup, Denmark) were used for detection using Lumi-Light^PLUS^ Western Blotting Substrate (Roche Diagnostics, Hilden, Germany). Membranes were probed with β-actin antibody (mouse, A5441; Sigma-Aldrich, St. Louis, MO) to confirm equal loading.

### Statistical analysis

#### MethylCap-seq

All methylation reads data were extracted using BioBix’ H2G2 browser (http://h2g2.ugent.be/biobix.html) for the broad promoter region (2000 bp upstream and 500 bp downstream of the transcription start site). Read counts were statistically compared between responder and non-responder groups using R/Bioconductor [29] package EdgeR [30], assuming that the data follow a negative binomial distribution, and ranked on *P* value.

Subsequently, integration of expression data was also performed using R-package LIMMA to find differentially expressed genes [31]. As an additional filter for further analysis, each candidate DMR had to be methylated (≥ 4 reads) in at least four samples of a specific response group. Given the fact that putatively relevant loci were selected based on both differential methylation and expression, and that several rounds of subsequent independent biological validation were performed, a relatively permissive error rate control cut-off (*P* = 0.05) was used for expression as well as validation.

#### Bisulfite pyrosequencing

Methylation percentage results were analyzed using statistical software IBM SPSS 21 (SPSS Inc., Chicago, IL) and a non-parametric statistical test (Mann–Whitney U test) was performed to find differences between responder and non-responder groups. *P* values of less than 0.05 were assumed to be statistically significant for all tests. To present data as a heatmap, all methylation percentage data were imported to Genesis software (Graz University of technology, genome.tugraz.at/genesis) for clustering and heatmap visualization.

### *In silico* validation of candidate markers

For prognostic validation of candidate gene methylation, methylation data of the AOCS and TCGA study groups was extracted and normalized as mentioned in ‘patient population involved’ Set 4 and Set 5, respectively. Low and high methylation cut-offs were based on median beta value. This resulted in 89 patients for PFS analysis (proxy for sensitivity to platinum containing chemotherapy) and 91 patients for OS analysis in AOCS data (Set 4). For the TCGA cohort (Set 5), we used 91 patients for PFS analysis and 105 patients for OS analysis. To handle the missing data, we used the listwise deletion methodology.

For marker expression, data (Set 6) was derived for analysis using KM plotter [21] in November 2015, in which we selected only advanced stage (3 and 4) HGSOC cancer patients with suboptimal debulking surgery, all of whom had received platinum therapy. This resulted in 200 patients for PFS and 208 patients for OS analysis using univariate Mantel–Cox log-rank survival analysis with *FZD10* probe (Probe ID: 219764_at), and 100 patients for PFS and 102 patients for OS analysis with *FAM83A* (Probe ID: 239586_at), *MYO18B* (Probe ID: 1554579_a_at), and *MKX* (Probe ID: 239468_at). With an expression range of probes for different genes, an auto cut-off value for PFS and OS analysis was used, based on computation of upper and lower quartiles with the default portal settings [21].

To review the gene expression of *FZD10* in other cancer types, we used the TCGA data from the TCGA FIREHOSE pipeline (http://gdac.broadinstitute.org/) [32]. To predict *FZD10* expression across 41 tumor types, we used their functional genomic mRNA (FGmRNA) profiles as described earlier [33, 34]. In this methodology, non-genetic transcriptional components were used as covariates to correct microarray expression data and the residual expression signal (i.e., FGmRNA profile) was found to capture the downstream consequences of genomic alterations on gene expression levels [33]. We quantified the percentage of samples across 41 tumor types with a significantly increased FGmRNA signal (i.e., a proxy for underlying gene amplification). For each of the 19,746 tumor samples, *FZD10* was marked as significantly amplified when the FGmRNA signal was above the 97.5th percentile threshold as defined in the non-cancer samples [33].

### In vitro experiments

Statistical significance was calculated by two-sided Student’s *t* test between two groups, unless otherwise mentioned in the figure legends. *P* values of less than 0.05 were defined as statistically significant for all tests.

## Results

### Discovery of DMRs in extreme chemoresponse HGSOC patients

**Fig. 1:**
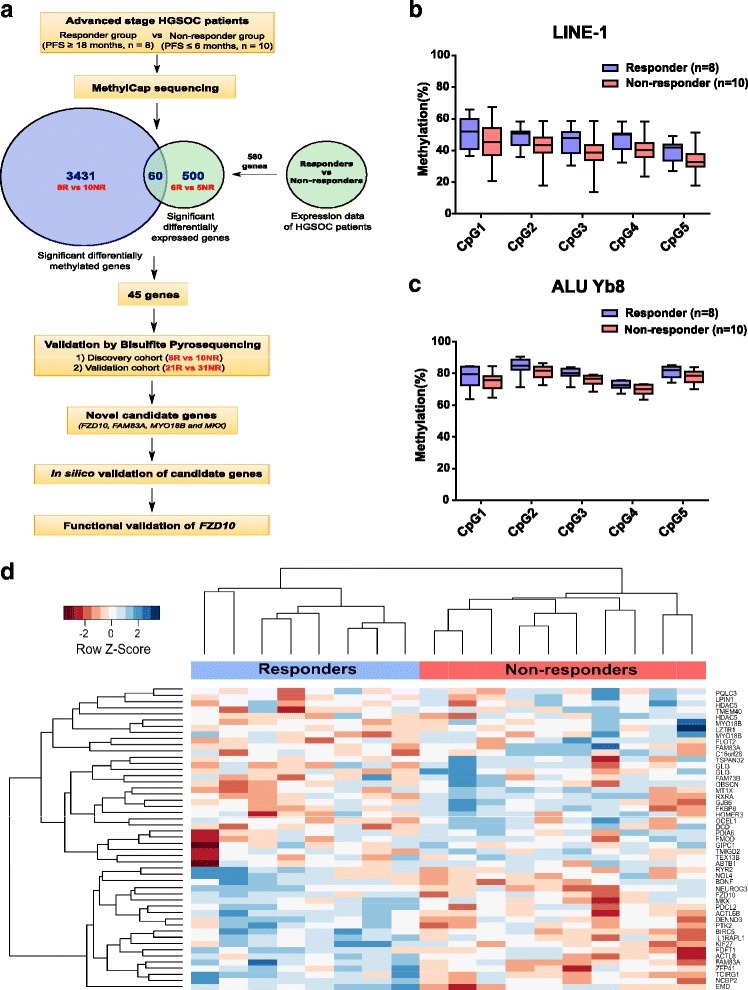
Identification of novel DNA methylation genes by using MethylCap-seq between extreme responder and non-responder HGSOC patients. **a** Experimental strategy to evaluate differential DNA methylation regions (DMRs) between extreme chemoresponse patient groups and their subsequent validation. **b** and **c** Bisulfite pyrosequencing for global methylation marker LINE-1 and ALU Yb6 in responder and non-responder groups showing similar global methylation level. Each bar represents average methylation in % ± SD of either responder (*n* = 8) or non-responder (*n* = 10) at a specific CpG site. **d** Hierarchical clustering of significant DMRs (49) in the responders (n = 8) and non-responders (*n* = 10) in the discovery set (Set 1)

In order to identify the DMRs in relation to platinum-based chemotherapy, we performed MethylCap-seq on primary tumor DNA of extreme responder (R = 8, PFS ≥ 18 months) and non-responder (NR = 10, PFS ≤ 6 months) HGSOC patients (Set 1) (Additional file 2: Table S1 and Fig. 1a). Upon normalization and bioinformatics analysis (see Methods), 4541 candidate DMRs comprising 3491 genes were identified (*P* < 0.05). Putative differences between the extreme responder and non-responder groups were not due to changes in global methylation, as demonstrated with the global methylation markers LINE-1 and ALU-Yb6 (Fig. 1b, c). The putative DMR data (3491 genes) was integrated with available RNA expression microarray data from 11 patients (Set 2: 6 responders and 5 non-responders) out of 18 that were used for MethylCap-seq. We found 560 genes that were putatively differentially expressed between the two extreme groups, of which 60 genes were both significantly differentially methylated and differentially expressed. To make sure that only the most relevant genes were selected, a DMR had to be methylated (e.g., four or more reads) in at least four samples in either the responder or the non-responder group. This resulted in 49 candidate DMRs comprising 45 genes (Additional file 5). Figure 1d shows clustering of these selected markers into two major sub-groups for chemoresponse with 29 hypomethylated and 20 hypermethylated DMRs in extreme responders in comparison with non-responders.

### *FZD10* was identified as the most differentially methylated gene between two chemoresponse-related groups

**Fig. 2:**
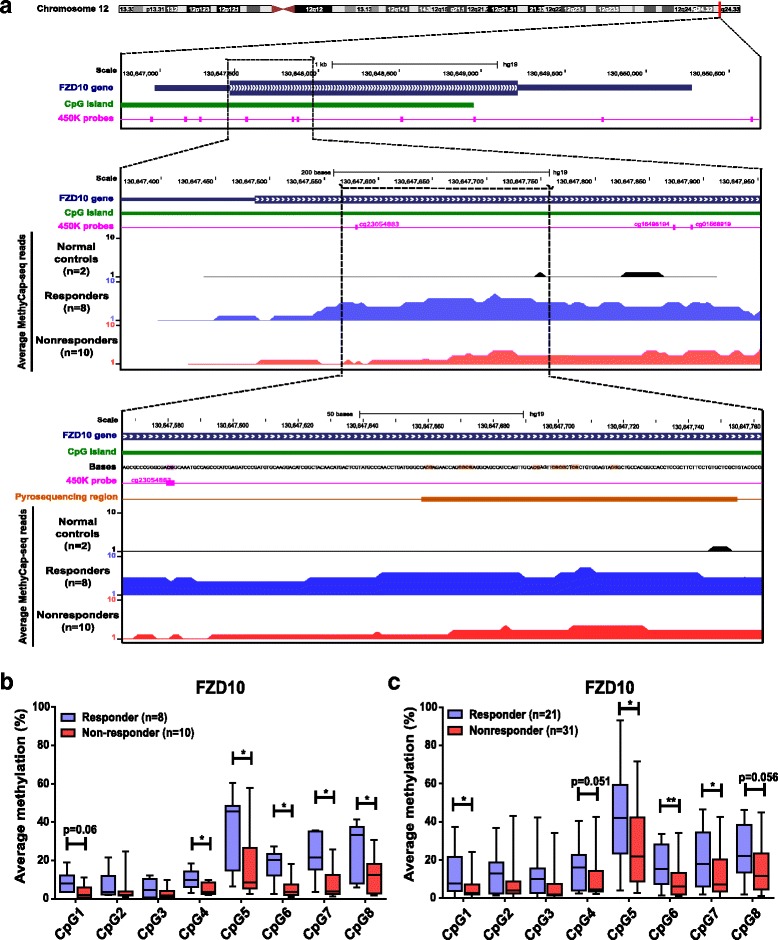
Bisulfite pyrosequencing verification and validation of MethylCap-seq data. **a** Schematic representation of the genomic region around the *FZD10* gene (chr12: q24.33, 130,647,000–130,650,400) as extracted from the UCSC browser (GRCh37/hg19 [63]; top of figure). The *FZD10* MethylCap-Seq region (middle of figure) located 130,647,308–130,647,889 (308–889 bp) downstream of the *FZD10* TSS, as retrieved from the map of the human methylome (BIOBIX, Dept. Mathematical Modelling, Statistics and Bioinformatics, Ghent, University of Ghent, Belgium, 2012, http://www.biobix.be). The reads retrieved by MethylCap-seq analysis comparing 2 normal control leucocytes (black color), 8 responders (blue color), and 10 non-responders (red color) HGSOC in this region. The known Infinium 450K probes (pink color) and CpG Island (green color) location as retrieved from the GSE42409 database [64]. The genomic region within the *FZD10* as sequenced by bisulfite pyrosequencing (orange color) (bottom of figure). **b** Verification of candidate chemoresponse methylation marker *FZD10* by bisulfite pyrosequencing in responder (blue bars, *n* = 8) and non-responder groups (red bars, *n* = 10) of discovery set showing significantly higher methylation in responder for *FZD10* compared to non-responder chemoresponse group. **c** Validation of *FZD10* in an independent external cohort of responder (blue bars, *n* = 21) and non-responders (red bar, *n* = 31). Each bar represents average methylation in % ± SD of either responder or non-responder at specific CpG sites. Mann–Whitney U test was performed, *P < 0.05, **P < 0.01

The 45 candidate genes were verified on the same samples used for MethylCap-seq by bisulfite pyrosequencing, as this assay is more quantitative and analyzes individual CpG sites. Pyrosequencing resulted in nine significantly differentially methylated genes: *FZD10*, *FAM83A*, *MYO18B*, *MKX*, *GLI3*, *TMIG2*, *TMEM40*, *NEUROG3*, and *HOMER3* (Table 1), of which *FZD10* exhibited the clearest effect. *FZD10* was more methylated in extreme chemoresponsive patients (significant (*P* < 0.05) in 5 of 8 CpG sites) (Fig. 2a, b). In addition, the methylation levels as quantified by bisulfite pyrosequencing significantly correlated with the reads of MethylCap-seq (Additional file 3: Figure S1A–D).

**Table 1:** Top genes that were verified using bisulfite pyrosequencing

Rank	Gene symbol	Gene Name	Total CpGs analyzed	Significant CpGs* Discovery Validation		Presence of CpG island	Correlation with mRNA expression
				(Set 1)	(Set 3)		
1	FZD10	Frizzled-homologue 10 (CD350 antigen)	8	5	4	Yes	Inverse
2	FAM83A	Family with sequence similarity 83, Member A (Tumor Antigen BJ-TSA-9)	7	5	4	No	Inverse
3	MYO18B	Myosin-XVIIIb	3	3	2	No	Inverse
4	MKX	Homeobox protein Mohawk	4	4	1	Yes	Inverse
5	GLI3	GLI Family Zinc Finger 3	6	5	0	Yes	Direct
6	TMIGD2	Transmembrane and immunoglobulin domain-containing 2 (CD28 homologue)	5	4	0	No	Direct
7	TMEM40	Transmembrane protein 40	6	6	0	No	Direct
8	NEUROG3	Neurogenin-3	16	13	0	Yes	Inverse
9	HOMER3	Homer protein homolog 3	7	6	0	No	Direct

*P < 0.05

The nine selected genes were then validated by bisulfite pyrosequencing in an external patient cohort of 21 extreme responders and 31 extreme non-responders (Set 3) with similar clinicopathological characteristics as the discovery patient cohort (Set 1) (Additional file 2: Table S1). This resulted in a final list of four candidate genes (*FZD10*, *FAM83A*, *MYO18B*, and *MKX*) with at least one significant CpG site in the external patient cohort (Table 1). Among these four candidate genes, *FZD10* contained the most methylated CpG sites, followed by *FAM83A*, *MYO18B*, and *MKX*. In agreement with the verification results, the same four CpGs in *FZD10* were significantly (*P* < 0.05) highly methylated (Fig. 2b, c) in the responder group. Likewise, we found significantly (*P* < 0.05) higher methylation of *MKX* in the responder group, whereas *FAM83A* and *MYO18B* showed higher methylation in the non-responder group.

### Candidate markers are epigenetically regulated genes

**Fig. 3:**
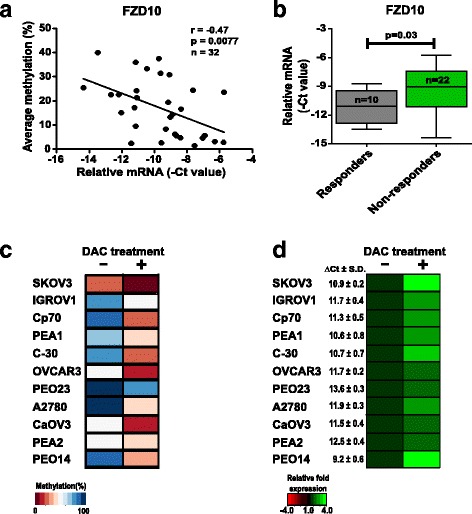
*FZD10* is an epigenetically regulated gene by DNA methylation. **a** Correlation analysis of average methylation as determined by bisulfite pyrosequencing and relative mRNA level of *FZD10* in external cohort patients (*n* = 32) showed significant inverse correlation between methylation and their correspondent expression using Pearson correlation testing. **b** qRT-PCR of *FZD10* was performed to determine relative mRNA levels in responder (*n* = 10) and non-responder HGSOC patient groups (*n* = 22). Heatmaps show average methylation percentage (**c**) and relative mRNA expression (**d**) of *FZD10* in various ovarian cancer cell lines (*n* = 11), treated with or without DAC for 72 h (DAC + or –). Most cell lines show DAC-induced demethylation (from blue to dark red, change in methylation percentage) with subsequent upregulation of mRNA (from black to green, relative fold expression). Relative gene expression of *FZD10* (∆Ct = Ct_FZD10_ – Ct_GAPDH_) for each untreated cell line is mentioned in front of the heatmap

To validate the impact of DNA methylation on expression of *FZD10*, *FAM83A*, *MYO18B*, and *MKX*, we determined the mRNA expression of available patient RNA samples for Set 3 using qRT-PCR. We found that the methylation levels of all four candidate markers were significantly inversely correlated with gene expression (Fig. 3a and Additional file 3: Figure S2A). Furthermore, *FZD10* gene expression was significantly lower in the extreme responder patient group compared to the non-responder group (Fig. 3b). Subsequently, we obtained similar results in a panel of 11 ovarian cancer cell lines, showing that high DNA methylation was related to low gene expression and vice versa (Fig. 3c, d and Additional file 3: Figure S2B). Moreover, after treatment with demethylating agent DAC, the DNA methylation level decreased with subsequent upregulation of expression of all four candidate genes in most cases (Fig. 3c, d and Additional file 3: Figure S2B). These results indicate that expression of all selected markers is epigenetically regulated in both ovarian cancer patients and cell lines.

### Predictive and prognostic impact of methylation and expression of candidate genes

**Fig. 4:**
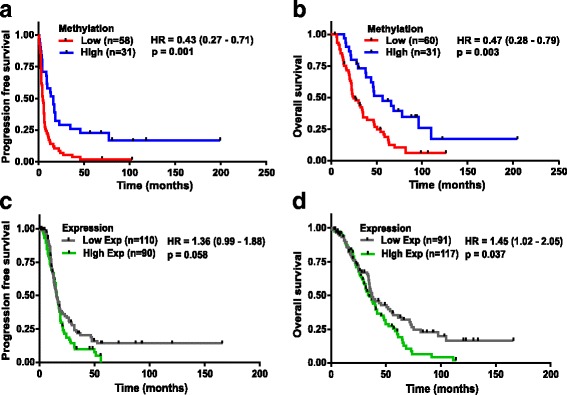
Predictive and prognostic evaluation of *FZD10* gene methylation and expression in HGSOC patients. **a**, **b** Kaplan–Meier plots showing PFS (**a**) and OS (**b**) for the two patient groups defined based on *FZD10* methylation using univariate Mantel–Cox log-rank survival analysis in HGSOC AOCS cohort (Set 4, n = 89 and n = 91, respectively). Average methylation β-value for ‘Low’ methylation group patients = 0.07 (0.04–0.09) and for ‘High’ methylation group patients = 0.14 (0.09–0.28). **c**, **d** Kaplan–Meier plots showing PFS (**c**) and OS (**d**) for the two patient clusters based on *FZD10* expression using univariate Mantel–Cox log-rank survival analysis in HGSOC cohorts (Set 6, n = 200 and n = 208, respectively)

After establishing the relationship between epigenetic silencing and its expression of validated markers, we investigated the potential predictive and prognostic value of marker methylation as well as expression. We used publicly available methylation and expression datasets (Sets 4, 5, and 6) with similar clinicopathological characteristics and treatment regimens as our discovery (Set 1) and validation cohorts (Set 3) without using the extreme chemoresponse criteria (PFS). After performing Cox regression analysis, we found that high methylation of *FZD10* was associated with better response to platinum containing chemotherapy of HGSOC patients (Set 4) as indicated by PFS (hazard ratio (HR) = 0.43 (0.27–0.71), *P* = 0.001) and improved OS (HR = 0.47 (0.28–0.79), *P* = 0.003) (Fig. 4a, b). In addition, we performed similar prognostic analysis on another independent methylation dataset from a HGSOC patient cohort (Set 5). Despite the low average methylation level of the *FZD10* methylation type I probe in Set 5 compared to the type II probe in Set 4 (methylation β-value of 0.022 vs. 0.09, *P* < 0.001), a trend was observed for high *FZD10* methylation and survival (PFS: HR = 0.68 (0.39–1.18), *P* = 0.17; OS: HR = 0.72 (0.44–1.21), *P* = 0.21). Moreover, average *FZD10* methylation of extreme responders in this cohort (Set 5) is higher than that of extreme non-responders (*P* = 0.059) (Additional file 3: Figure S3A–C). An opposite relation was found when the predictive and prognostic value of *FZD10* gene expression levels was determined. High *FZD10* gene expression (Set 6) was associated with a worse response and prognosis (PFS: HR = 1.36 (0.99–1.88), *P* = 0.058; OS: HR = 1.345 (1.02–2.05), *P* = 0.037 (Fig. 4c, d)).

In addition, no effect of *FAM83A* methylation on the survival of HGSOC patients was observed (Additional file 3: Figure S4A, B). However, we found that high *FAM83A* expression was associated with a better prognosis (OS: HR = 0.52 (0.34–0.86), *P* = 0.01; Additional file 3: Figure S4D). Furthermore, *MYO18B* and *MKX* methylation were associated with patient survival. High *MYO18B* methylation showed a trend towards better response (PFS: HR = 0.67 (0.43–1.04), *P* = 0.077) but no association with overall survival (Additional file 3: Figure S5A, B). Likewise, high methylation of *MKX* was associated with better response and prognosis (PFS: HR = 0.59 (0.38–0.91), *P* = 0.018; OS: HR = 0.57 (0.35–0.93), *P* = 0.024; Additional file 3: Figure S6A, B).

To investigate whether DNA methylation is an independent prognostic factor or not, we performed uni- and multivariate analyses on age, stage, and all four methylation markers using the external methylation dataset 4 (*n* = 91). We found that neither age nor stage was significantly associated with PFS in univariate analysis (Additional file 2: Table S3). Age was found to be significantly associated with OS in multivariate analysis (HR = 0.97 (0.94–1.00), *P* = 0.040). Notably, in multivariate analysis high *FZD10* and *MKX* methylation was found to be significantly associated with better PFS (for *FZD10*: HR = 0.39 (0.23–0.65), *P* = 0.003; for *MKX*: HR = 0.49 (0.31–0.77), *P* = 0.002) as well as OS (for *FZD10*: HR = 0.40 (0.24–0.68), *P* = 0.001; for *MKX*: HR = 0.46 (0.28–0.75), *P* = 0.002; Additional file 2: Table S3). In conclusion, these results demonstrate that, among all candidate markers, only for *FZD10* both methylation and expression have prognostic value for response to platinum-based chemotherapy in advanced stage HGSOC patients. Moreover, *FZD10* methylation also has independent prognostic value. Hence, we chose *FZD10* for further functional validation on ovarian cancer cell lines.

### Downregulation of *FZD10* enhances cisplatin-induced cell growth inhibition and apoptosis in ovarian cancer cell lines

**Fig. 5:**
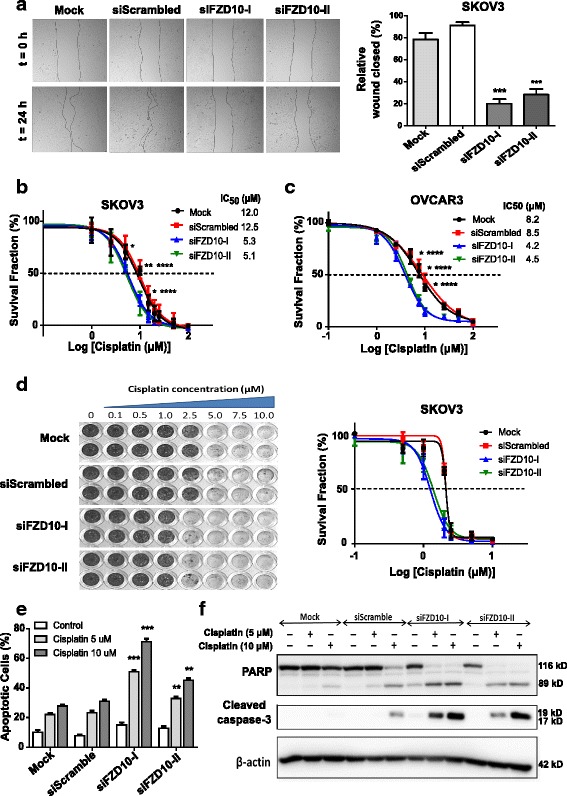
*FZD10* silencing shows low migratory phenotype in the ovarian cancer cell lines and sensitizes towards cisplatin treatment. **a** Representative microphotographs (4× magnification) for wound healing assay on FZD10 siRNA-treated SKOV3 cells for T = 0 and T = 24 h, along with the quantification of relative wound. Each bar represents % of wound closed ± SD from three independent experiments. ***P < 0.001 for FZD10 siRNA-treated cells in comparison to the scrambled siRNA (siScrambled), by Student *t* test. **b, c** Short-term MTT survival assay on siRNA-treated SKOV3 and OVCAR3 cells and relative survival in the presence of cisplatin at indicated concentration after 96 h. *P < 0.05; **P < 0.01 for siFZD10-I and ****P < 0.05 for siFZD10-II relative to expression in siScrambled control, Student *t* test. IC_50_ was calculated and mentioned for each group in the inset. **d** Representative photograph and quantification of long-term survival assay of SKOV3 cells treated with FZD10 siRNAs. Cells were grown in the absence or presence of cisplatin at the indicated concentrations for 10 days. **e** Determination of apoptotic cells in SKOV3 cells treated with siScrambled or FZD10 siRNAs (siFZD10-I or siFZD10-II). After cisplatin treatment for 48 h, apoptosis induction was analyzed by fluorescence microscopy on acridine orange-stained cells. Each bar represents % of apoptotic cells ± SD from three or four independent experiments. **P < 0.01, *** *P* < 0.001 for either siFZD10-I or siFZD10-II with respect to their siScrambled treated cells. **f** Protein levels of cleaved PARP and caspase 3 in SKOV3 cells transiently transfected with either FZD10 along with treatment of cisplatin for 24 h with the indicated concentrations

FZD10 has been described as a functionally relevant WNT pathway receptor in several cancer types [35–38]. FZD10 expression has not been previously related to cisplatin sensitivity. To study the functional role of FZD10 in ovarian cancer, *FZD10* gene expression was transiently downregulated in SKOV3 and OVCAR3 cells using two independent *FZD10* targeted siRNAs. We found 70–80% down-regulation of mRNA levels in SKOV3 and 50–60% down-regulation in OVCAR3 for up to 2–4 days (Additional file 3: Figure S7A). Transient silencing of *FZD10* did not affect the proliferation rate of cell lines when compared to scrambled siRNA controls (Additional file 3: Figure S7B). However, we found significant reduction (*P* < 0.001) in the migratory potential of FZD10 siRNA-treated cells as compared with scrambled and mock controls (Fig. 5a and Additional file 3: Figure S7C).

Short-term survival assays of 4 days showed 2 to 2.5 times greater sensitivity (*P* < 0.05) to cisplatin in FZD10 siRNA-treated cells (SKOV3, OVCAR3, C-30, and PEA2) compared to the scrambled siRNA or non-transfected control counterparts (Fig. 5b, c, Additional file 3: Figure S7D, E). Furthermore, similar significant cisplatin sensitizing effects of transient silencing of FZD10 were observed in long-term survival assays of 10 days in the SKOV3 cell line (Fig. 5d).

To gain more insight into the cisplatin sensitizing effect of FZD10 downregulation, we performed apoptosis staining and analyzed the early apoptotic markers PARP and caspase 3. A significant increment in apoptosis by 15–40% (*P* < 0.001) after exposure for 48 h to various concentrations of cisplatin was observed in *FZD10*-silenced SKOV3 cells compared to scrambled siRNA and control cells (Fig. 5e). Apoptosis results were confirmed by an increase in cleaved PARP and cleaved caspase 3 protein levels (Fig. 5f). Likewise, downregulation of FZD10 in OVCAR3 cells resulted in cisplatin sensitization in comparison to cisplatin-treated scrambled siRNA and mock controls (Additional file 3: Figure S7E).

Taken together, these results prove that FZD10 is a determinant of cisplatin sensitivity of ovarian cancer cells.

## Discussion

Despite increased understanding of the molecular characteristics of ovarian cancers, no validated clinically relevant markers for platinum chemoresponse in ovarian cancer are currently available. In this study, we identified novel epigenetically-regulated chemoresponse markers for extreme HGSOC platinum responder and non-responder patients by genome-wide DNA methylation-enriched sequencing (MethylCap-seq). We discovered that four genes (*FZD10*, *FAM83A*, *MYO18B*, and *MKX*) were differentially methylated and expressed between extreme responders and non-responders. In silico analysis on publicly available DNA methylation and expression datasets of unselected advanced stage HGSOC patients showed that DNA methylation of *FZD10* and *MKX* was independently prognostic for improved chemoresponse, as reflected by PFS. In accordance with high *FZD10* methylation, low *FZD10* expression was associated with a better chemotherapy response and overall survival. Functional analyses of FZD10 established its clear role in cisplatin sensitivity and migration of ovarian cancer cells.

Previously, the identification of epigenetic platinum chemoresponse markers in HGSOC was performed on customized or commercially available methylation array-based platforms with a limited number of CpG probes [14–16]. In the current study, the overall genome-wide DNA methylation profile information was obtained using MethylCap-seq. A recent study has shown that MethylCap-seq technology is a promising unbiased approach for genome-wide DNA methylation profiling that outperforms other methylated DNA capturing techniques [39]. Furthermore*,* MethylCap-seq has comparable coverage of CpG sites at promoter region and CpG islands to whole-genome bisulfite sequencing [40]. Moreover, MethylCap-seq has been shown to be sensitive in various cancer types, including head and neck, non-small cell lung cancer and cervical cancer [24, 41–44]. Thus far, only one study reported a comprehensive analysis on a large ovarian cancer patient cohort (*n* = 101; 75 malignant, 20 benign and 6 normal) using MethylCap-seq [45]. The DMRs of malignant tumors were compared to benign or normal samples. However, platinum chemotherapy response was not included in the analysis.

By combining the genome-wide methylation and expression data of HGSOC patients and subsequent validations, we identified four novel epigenetically regulated candidate genes (*FZD10*, *FAM83A*, *MYO18B*, and *MKX*) that were differentially methylated between extreme responders and non-responders. In silico analysis of unselected advanced stage HGSOC patients showed that DNA methylation of *FZD10* and *MKX* was independently associated with a better chemoresponse. Because *FZD10* was the only gene showing both methylation and expression to have prognostic value for the response to platinum-based chemotherapy, this study focused further on *FZD10* for functional validation. However, it is possible that the other genes also play a role in platinum chemoresponse in HGSOC. FAM83A, also known as BJ-TSA-9, is highly expressed in lung cancer [46] and is highly amplified in many cancer types including breast, ovarian, lung, liver, prostate, and pancreas [47]. Recently, FAM83A has been found to be a key mediator of resistance to many EGFR tyrosine-kinase inhibitors in breast cancer by causing phosphorylation of c-RAF and PI3K p85, thus promoting proliferation of and invasion by breast cancer cells [48]. *MYO18B* has been reported to be hypermethylated in ovarian cancer and important for carcinogenesis [11]. MKX (IRXL1) is known for its role in muscle development [49]; recently, it has been identified as an epigenetically regulated gene by microRNA 662 in ovarian cancer [50], but its role in ovarian cancer is unknown. Interestingly, we previously identified *MKX* hypermethylation as an early detection biomarker for cervical cancer [24]. None of these four genes has been associated with chemo-resistance or sensitivity in HGSOC, indicating that all four might be novel chemoresponse markers for platinum-based chemotherapy.

FZD10 is a member of the Frizzled family of seven-transmembrane WNT signaling receptors [51]. FZD10 overexpression has been reported in primary cancers such as colon, sarcomas, endometrial, gliomas, and ovarian cancer [35–38, 46, 51] (Additional file 3: Figure S8). FZD10 is assumed to play a role in invasion and metastasis via either the canonical (in colon, endometrial, and breast cancer) or non-canonical WNT pathway (in sarcomas) in a cancer type-dependent manner [36, 38, 52, 53]. In the present study, we showed that downregulation of FZD10 causes a less migratory phenotype in ovarian cancer cell lines. Moreover, using a FZD10 silencing approach, we showed that FZD10 expression is not only involved in promoting migration, but also causally related to cisplatin resistance of ovarian cancer cells. In agreement with these in vitro results, we found that high *FZD10* expressing HGSOC tumors were worse responders to platinum-based chemotherapy. In a study on ovarian vascular markers, Buckanovich et al. [54] showed that low expression of *FZD10* in ovarian cancer is significantly associated (*P* = 0.001) with better prognosis, which is in line with our findings of significantly high *FZD10* methylation and low *FZD10* expression in the responder patient group in comparison to non-responders. In addition, our previously published study [18] on global gene expression analysis of HGSOC patients (*n* = 156) also showed that high *FZD10* expression was associated with poor overall survival (HR 1.57, *P* = 0.0086). Since *FZD10* expression is absent or hardly detectable in any normal organs except placenta [55] and highly expressed in ovarian cancer (Additional file 3: Figure S8), our results indicate that *FZD10* is an interesting therapeutic target in ovarian cancer. Furthermore, considering the expression of *FZD10* in other tumor types (Additional file 3: Figure S8), *FZD10* may play a role in other tumor types like uterine corpus endometrial cancer and cervical cancer, which are treated with platinum-based chemotherapy often in combination with radiotherapy. Notably, FZD10 has been shown to be a therapeutic target in synovial sarcomas; these sarcomas displayed attenuated growth when targeted by a polyclonal FZD10 antibody [52]. In addition, a radiation-labeled humanized monoclonal antibody against FZD10 (*OTSA101*) has been recently developed, and is currently in phase I clinical trials for synovial sarcoma [56]. This approach might also be interesting in the context of chemoresistant ovarian cancer.

Although HGSOC is known for bearing mutations in a limited number of genes, aberrant DNA methylation has been found, which might have an effect on platinum-based chemotherapy response [19, 45, 57]. In addition to the four novel epigenetically regulated genes, we also found other known genes that have been reported for chemoresponse in ovarian cancer or other cancer types. For instance, Survivin (*BIRC5*) was among the top 45 gene list from our analysis and has been reported to be involved in platinum sensitivity in HGSOC [58]. Another gene from our analysis, *GLI3* (a gene of Hedgehog signaling) has been mentioned as being epigenetically regulated and linked with platinum response in HGSOC [45]. However, *GLI3* could only be verified with pyrosequencing but failed during further validation in our study (Table 1). Previous reports described several hypermethylated genes that we also found in our initial MethylCap-seq analysis list (4541 DMRs) (Fig. 1a). For instance, *BRCA1* hypermethylation was found to be positively associated with chemosensitivity [6, 19, 59]. Furthermore, hypermethylation of other DNA damage repair pathway-related genes, like *GSTP1*, *FANCF*, and *MGMT*, has been described to be positively associated with chemosensitivity in ovarian cancer patients [13, 60]. Hypermethylation of genes like *ASS1*, *MLH1*, and *MSX1*, and WNT pathway-related genes including *DVL1*, *NFATC3*, and *SFRP5* was related to poor outcome of ovarian cancer patients treated with platinum-based chemotherapy [13, 14, 61, 62]. These genes were omitted from the gene list, since we only included genes that were significantly differentially methylated as well as expressed between responders and non-responders.

## Conclusions

By applying genome-wide integrated methylome analysis on extreme chemoresponsive HGSOC patients, we identified novel clinically relevant, epigenetically-regulated markers of platinum-sensitivity in HGSOC patients. Resulting candidate genes were successfully validated in an independent patient cohort. Consequently, we found *FZD10* as a functionally validated novel methylated gene for platinum-based chemoresponse in HGSOC patients. The clinical potential of these markers in predictive and therapeutic approaches has to be further validated in prospective studies.

## Additional files

Additional file 1: REporting recommendations for tumor MARKer prognostic studies (REMARK) Check-list. (DOC 61 kb)

Additional file 2: Table S1. Patient characteristics of all patient cohorts (Set 1–6) for methylation and expression analysis. **Table S2.** Ovarian cancer cell lines and their culture conditions used in this study. **Table S3.** Result of uni- and multivariate survival analysis of external methylation Set 4 (*n* = 91). (DOCX 33 kb)

Additional file 3: Figure S1. Spearman correlation of MethylCap-seq reads and methylation percentage obtained with bisulfite pyrosequencing for *FZD10*, *FAM83A*, *MYO18B* and *MKX*. **Figure S2**. Correlation analysis of average methylation as determined by bisulfite pyrosequencing and microarray-based expression levels of *FAM83A*, *MYO18B* and *MKX*. **Figure S3**. Kaplan–Meier plots showing PFS and OS for the two user-defined patient groups based on *FZD10* methylation levels on the HGSOC TCGA cohort (Set 5). **Figure S4-S6**. Kaplan–Meier plots showing PFS and OS association for the two user-defined patient groups based on methylation (Set 4) and expression (Set 6) of *FAM83A*, *MYO18B* and *MKX*. **Figure S7**. Functional validation of FZD10 in the ovarian cancer cell lines. **Figure S8**. Global relative expression of *FZD10* in different types of cancer based on the TCGA data. (PDF 1218 kb)

Additional file 4: Primers sequences for bisulfite pyrosequencing and qRT-PCR, and FZD10 siRNA duplex sequences. (XLSX 11 kb)

Additional file 5: Top 45 genes (49 DMRs) for verification along with their MethylCap-seq data and corresponding expression data. (XLSX 40 kb)

## Abbreviations

DAC: decitabine (2′-deoxy-5-azacytidine)
DMRs: differentially methylated regions
FGmRNA profile: functional genomic mRNA profile
FIGO: International Federation of Gynecology and Obstetrics
HGSOC: high-grade serous ovarian cancer
MethylCap-seq: enrichment of methylated DNA using the methyl-CpG binding domain of MeCP2 protein followed by next generation-sequencing
MTT: 3-(4, 5-dimethythiazol-2-yl)-2,5-diphenyltetrazolium bromide
OS: overall survival
PFS: progression-free survival
qRT-PCR: quantitative reverse transcriptase polymerase chain reaction
SD: standard deviation
UMCG: University Medical Center Groningen
