# Supplementary material for: Methylome analysis of extreme chemoresponsive patients identifies novel markers of platinum sensitivity in high-grade serous ovarian cancer
Source: BMC Med. 2017 Jun 23;15:116. doi: 10.1186/s12916-017-0870-0 (PMC5481993; doi:10.1186/s12916-017-0870-0)
Supplement: Supplementary file 1 — REporting recommendations for tumor MARKer prognostic studies (REMARK) Check-list. (DOC 61 kb) [file 12916_2017_870_MOESM1_ESM.doc]

| **Item to be reported** | | **Explanation and reference in the manuscript** |
| --- | --- | --- |
| **INTRODUCTION** | |  |
| 1 | State the marker examined, the study objectives, and any pre-specified hypotheses. | Page no. 5, Lines 106-109 |
| **MATERIALS AND METHODS** | |  |
| *Patients* | |  |
| 2 | Describe the characteristics (e.g., disease stage or co-morbidities) of the study patients, including their source and inclusion and exclusion criteria. | In the ‘Methods’ section, under ‘Patient population involved’ including Set 1- Set 5, (Page no. 6-8); Supplementary Table S1 |
| 3 | Describe treatments received and how chosen (e.g., randomized or rule-based). | Supplementary Table S1; randomization is not applicable |
| *Specimen characteristics* | |  |
| 4 | Describe type of biological material used (including control samples) and methods of preservation and storage. | Frozen tumor tissue, In the ‘Methods’ section, under ‘Patient population involved’ including Set 1-Set 5 (Page no. 6-8) |
| *Assay methods* | |  |
| 5 | Specify the assay method used and provide (or reference) a detailed protocol, including specific reagents or kits used, quality control procedures, reproducibility assessments, quantitation methods, and scoring and reporting protocols. Specify whether and how assays were performed blinded to the study endpoint. | MethylCap-seq (references 23, 24), bisulfite pyrosequencing (reference 26) and quantitative reverse transcriptase PCR (qRT-PCR, reference 26) were used (In ‘Methods’ section, page no. 9 -11)  The researcher was not blinded to the group allocation. However, as all samples are anonymous and clinical data was merged afterwards, this is close to blindness. This is not specifically described in the manuscript, but is common scientific behavior. |
| *Study design* | |  |
| 6 | State the method of case selection, including whether prospective or retrospective and whether stratification or matching (e.g., by stage of disease or age) was used. Specify the time period from which cases were taken, the end of the follow-up period, and the median follow-up time. | This is an retroprospective study with specified inclusion criteria mentioned in the ‘Methods’ sections, under ‘Patient population involved’ including Set 1-Set 5 (Page no. 6-8) |
| 7 | Precisely define all clinical endpoints examined. | Progression free survival (PFS) and overall survival (OS) |
| 8 | List all candidate variables initially examined or considered for inclusion in models. | Advanced stage, high grade, serous histological subtype, with residual diseases, treated with platinum-based chemotherapy and availability of PFS and OS data |
| 9 | Give rationale for sample size; if the study was designed to detect a specified effect size, give the target power and effect size. | No sample size calculation was performed with following explanations:  1) For discovery set (Set 1):  (i) Power calculations are impossible due to the moderated nature of the analysis (i.e. all loci are necessarily considered together for variance estimation). (ii) This approach was only intended to identify candidate methylated gens for subsequent validation.  2) For validation set (Set 3): Given the observed significant results for the verification experiment (see results in Fig 2B), the use of similar sample sizes for the validation experiment should lead to overall similar significant results if the observed variances are equal. Though formal power analysis is complicated due to the fact that non-parametric statistical tests were used, it is clear that a validation experiment with almost three times the number of responders and non-responders, as performed here, should be sufficiently powered to identify (at least) the same differences.  3) In silico analysis (Set 4 and 5): We used all publicly available data with clinicopathological details of patients. |
| *Statistical analysis methods* | |  |
| 10 | Specify all statistical methods, including details of any variable selection procedures and other model-building issues, how model assumptions were verified, and how missing data were handled. | All statistical methodology used in this study is described in the ‘Methods’ section under ‘Statistical analysis’ (page 13-15)  Estimation of variation within each group is not applicable, because this study first aimed to identify novel methylation markers, which were subsequently validated in patient material and cell lines. For handling the missing data, we used listwise deletion methodology. |
| 11 | Clarify how marker values were handled in the analyses; if relevant, describe methods used for cutpoint determination. | For *in silico* analysis median methylation or expression was used as cut-off point. Described in the ‘Methods’ section ‘Statistical analysis’ (page 13-15) |
| **RESULTS** | |  |
| *Data* | |  |
| 12 | Describe the flow of patients through the study, including the number of patients included in each stage of the analysis (a diagram may be helpful) and reasons for dropout. Specifically, both overall and for each subgroup extensively examined report the numbers of patients and the number of events. | In the ‘Methods’ section, under ‘Patient population involved’ including Set 1- Set 5, (Page no. 6-8); Supplementary Table S1 |
| 13 | Report distributions of basic demographic characteristics (at least age and sex), standard (disease-specific) prognostic variables, and tumor marker, including numbers of missing values. | This is shown in supplementary Table S1 |
| *Analysis and presentation* | |  |
| 14 | Show the relation of the marker to standard prognostic variables. | This is described in the ‘Result’ section under ‘Predictive and prognostic impact of methylation and expression of candidate genes’ (page 17-18) |
| 15 | Present univariable analyses showing the relation between the marker and outcome, with the estimated effect (e.g., hazard ratio and survival probability). Preferably provide similar analyses for all other variables being analyzed. For the effect of a tumor marker on a time-to-event outcome, a Kaplan-Meier plot is recommended. | This is mentioned in the ‘Result’ section under ‘Predictive and prognostic impact of methylation and expression of candidate genes’ (page 17-18) and Fig. 4, Supplementary Fig. S3-S5 |
| 16 | For key multivariable analyses, report estimated effects (e.g., hazard ratio) with confidence intervals for the marker and, at least for the final model, all other variables in the model. | This is mentioned in the ‘Result’ section under ‘Predictive and prognostic impact of methylation and expression of candidate genes’ (page 17-18) |
| 17 | Among reported results, provide estimated effects with confidence intervals from an analysis in which the marker and standard prognostic variables are included, regardless of their statistical significance. | This is mentioned in the ‘Result’ section under ‘Predictive and prognostic impact of methylation and expression of candidate genes’ (page 17-18) and Fig. 4, Supplementary Fig. S3-S5 |
| 18 | If done, report results of further investigations, such as checking assumptions, sensitivity analyses, and internal validation. |  |
| **DISCUSSION** | |  |
| 19 | Interpret the results in the context of the pre-specified hypotheses and other relevant studies; include a discussion of limitations of the study. | The interpretation of the results has been incorporated in the ‘Discussion’, Page no. 20-23 including mentioning the limitations e.g. Some interesting genes were omitted from the gene list, since we only included genes that were significantly differentially methylated as well as expressed between responders and non-responders. |
| 20 | Discuss implications for future research and clinical value. | Implications are discussed, in the ‘Discussion’; Page no. 22 Lines 550-552 and in the ‘Conclusions’ section of manuscript |
