# Supplementary material for: Methylome analysis of extreme chemoresponsive patients identifies novel markers of platinum sensitivity in high-grade serous ovarian cancer
Source: BMC Med. 2017 Jun 23;15:116. doi: 10.1186/s12916-017-0870-0 (PMC5481993; doi:10.1186/s12916-017-0870-0)
Supplement: Supplementary file 2 — Patient characteristics of all patient cohorts (Set 1–6) for methylation and expression analysis. Table S2. Ovarian cancer cell lines and their culture conditions used in this study. Table S3. Result of uni- and multivariate survival analysis of external methylation Set 4 (n = 91). (DOCX 33 kb) [file 12916_2017_870_MOESM2_ESM.docx]

| * | Histology of cell lines is based on Beaufort et al^@^. |
| --- | --- |
| ATCC | American type culture collection |
| ^#^ | Imperial College London, London, UK |
| ^$^ | Fox Chase Cancer Center, Philadelphia, USA |
| HGSOC | High grade serous ovarian cancer |
| -/- | Homozygous deletion |
| Mut | Mutation |
| WT | Wild type |

**^@^** Beaufort CM, Helmijr JCA., Piskorz AM, Hoogstraat M, Ruigrok-Ritstier K, Besselink N, et al. Ovarian Cancer Cell Line Panel (OCCP): Clinical Importance of In Vitro Morphological Subtypes. PLoS One. 2014;9:e103988

|  |  |
| --- | --- |

*included for multivariate analysis
