# Supplementary material for: Methylome analysis of extreme chemoresponsive patients identifies novel markers of platinum sensitivity in high-grade serous ovarian cancer
Source: BMC Med. 2017 Jun 23;15:116. doi: 10.1186/s12916-017-0870-0 (PMC5481993; doi:10.1186/s12916-017-0870-0)
Supplement: Supplementary file 3 — Spearman correlation of MethylCap-seq reads and methylation percentage obtained with bisulfite pyrosequencing for FZD10, FAM83A, MYO18B and MKX. Figure S2. Correlation analysis of average methylation as determined by bisulfite pyrosequencing and microarray-based expression levels of FAM83A, MYO18B and MKX. Figure S3. Kaplan–Meier plots showing PFS and OS for the two user-defined patient groups based on FZD10 methylation levels on the HGSOC TCGA cohort (Set 5). Figure S4-S6. Kaplan–Meier plots showing PFS and OS association for the two user-defined patient groups based on methylation (Set 4) and expression (Set 6) of FAM83A, MYO18B and MKX. Figure S7. Functional validation of FZD10 in the ovarian cancer cell lines. Figure S8. Global relative expression of FZD10 in different types of cancer based on the TCGA data. (PDF 1218 kb) [file 12916_2017_870_MOESM3_ESM.pdf]

Figure S1

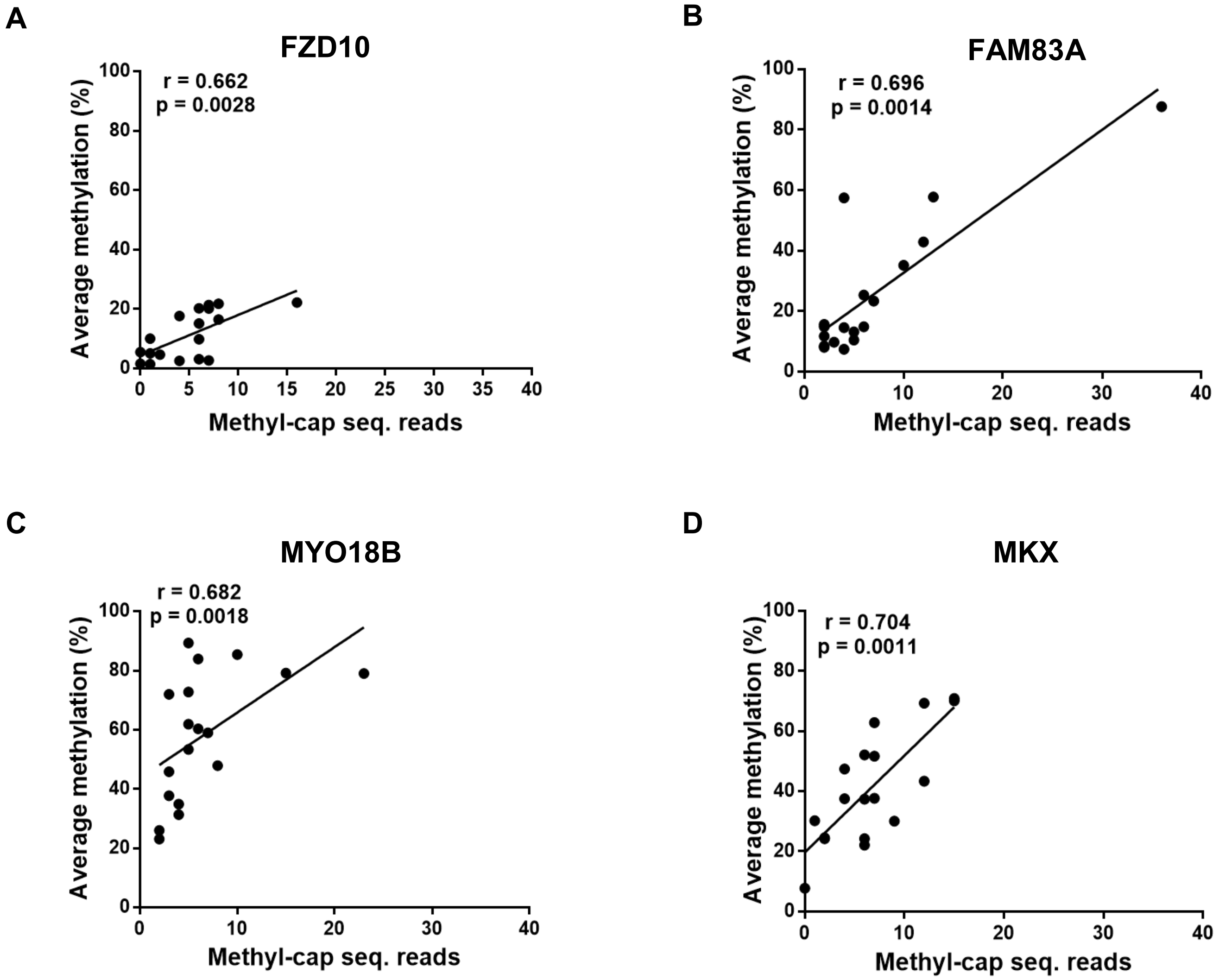

Figure S1. Spearman correlation of MethylCap-seq reads and methylation percentage obtained with bisulfite pyrosequencing for **A)** *FZD10*, **B)** *FAM83A*, **C)** *MYO18B* and **D)** *MKX* showing successful verification of MethylCap-seq data by pyrosequencing for discovery set 1 (n=18).

Figure S2

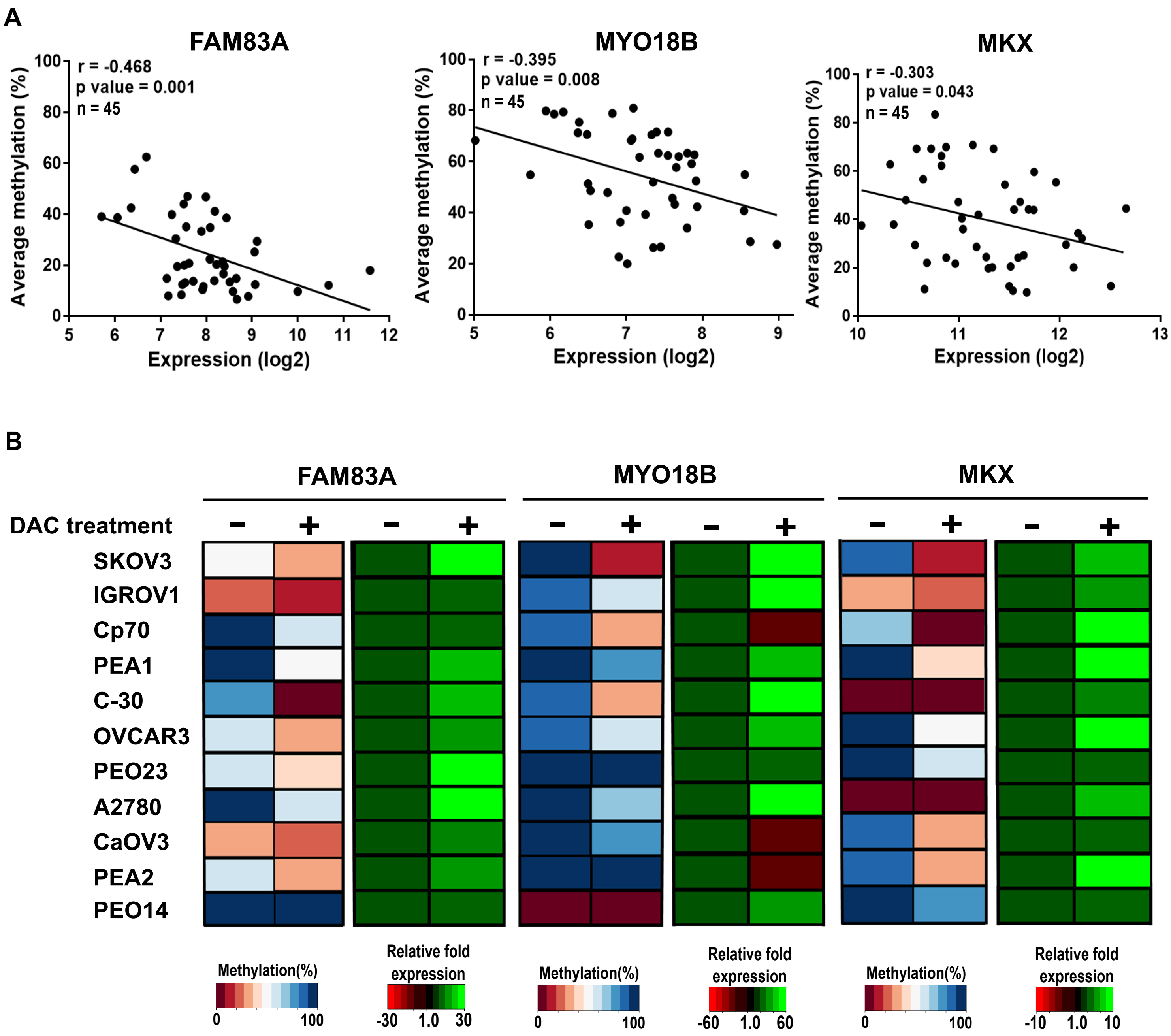

Figure S2. A) Correlation analysis of average methylation as determined by bisulfite pyrosequencing and microarray-based expression levels [18] of *FAM83A*, *MYO18B* and *MKX* in HGSOC patients (n=45) showing a significant inverse correlation between methylation and their correspondent expression using Pearson correlation testing. **B)** Heat maps of average methylation percentage and relative mRNA expression of *FAM83A*, *MYO18B* and *MKX* in various ovarian cancer cell lines (n=11), treated with or without demethylating agent 5-aza-2'-deoxycytidine (DAC) for 72 hrs (DAC + or -) showing demethylation (from blue to dark red) in most of the cell lines with subsequent upregulation of mRNA (from black to green).

Figure S3

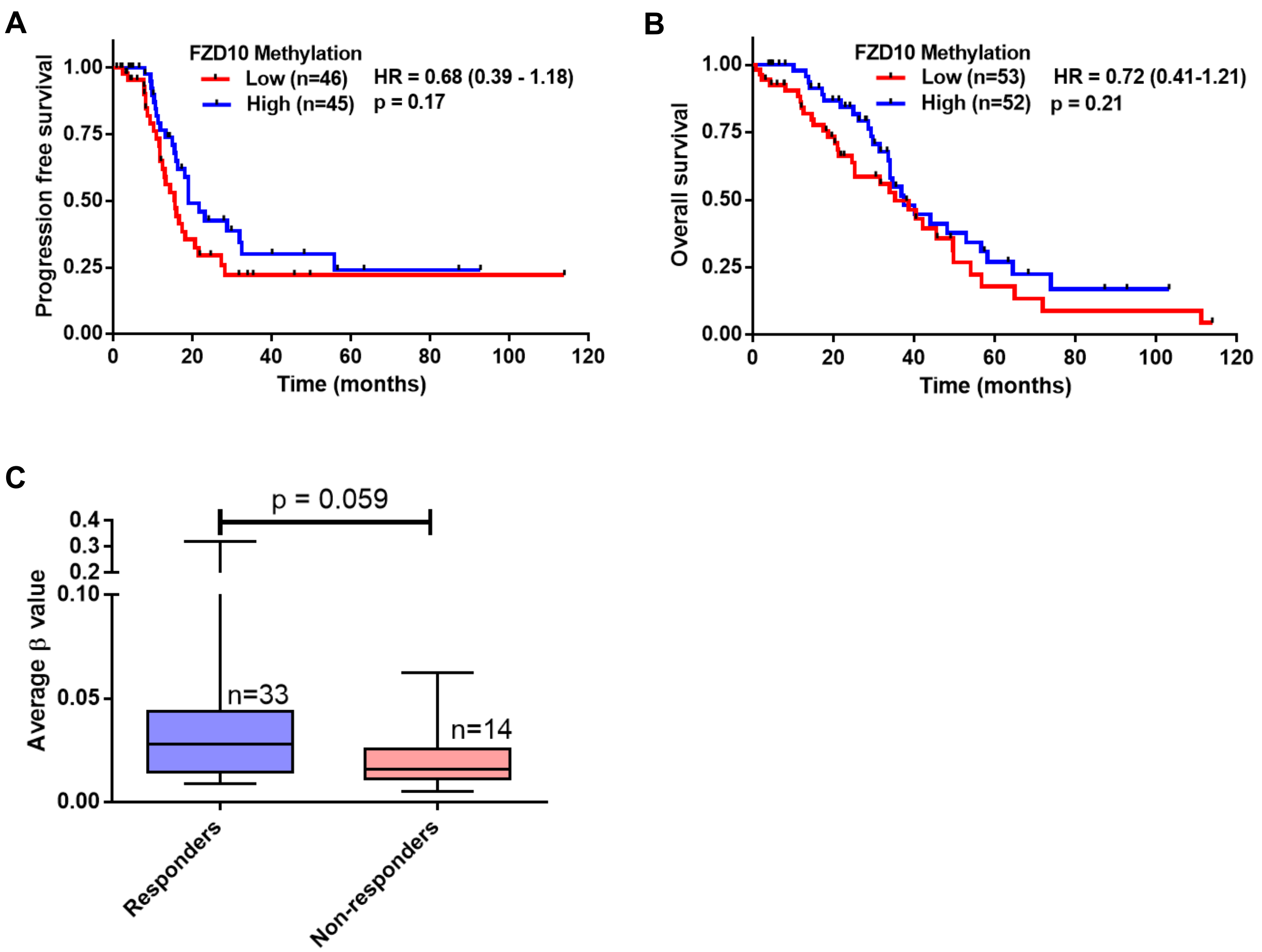

Figure S3. A-B) Kaplan–Meier plots showing PFS (**A**) and OS (**B**) for two user-defined patient groups based on *FZD10* methylation levels using univariate Mantel-Cox log-rank survival analysis on the HGSOC TCGA cohort (Set 5, n=91 and n=105, respectively). Average methylation β-value for 'Low' methylation group patients = 0.01 (0.01-0.02) and for 'High' methylation group patients = 0.06 (0.02-0.61). **C**) Average methylation β-value of *FZD10* in extreme responder (blue bars, n=33) and non-responder groups (red bars, n=14) of the TCGA cohort (Set 5) shows higher *FZD10* methylation in the extreme responder compared to the extreme non-responder group.

Figure S4

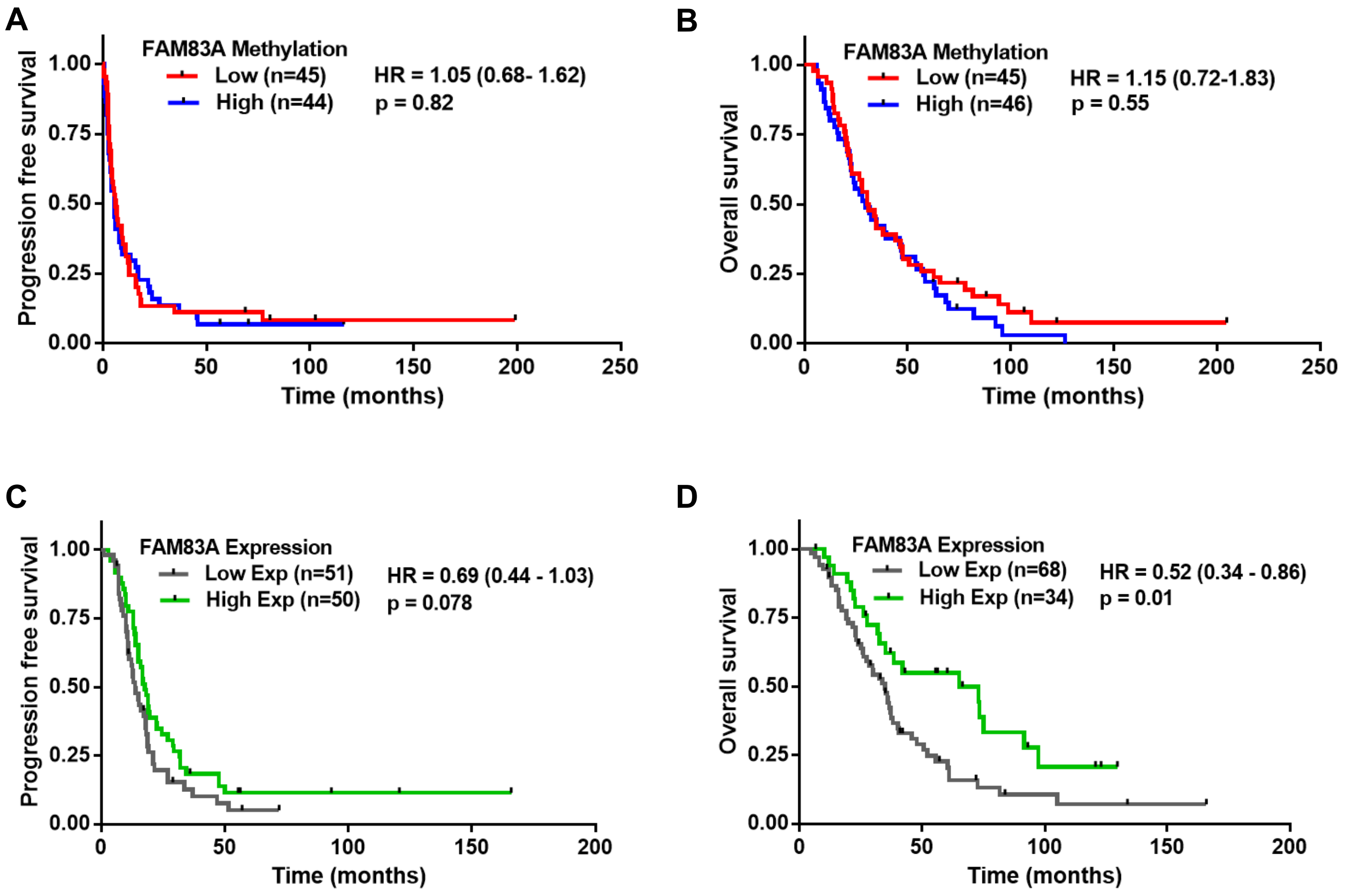

Figure S4. A-B) Kaplan–Meier plots showing PFS (**A**) and OS (**B**) for the two user-defined patient groups based on *FAM83A* methylation using univariate Mantel-Cox log-rank survival analysis in HGSOC cohorts (Set 4, n=89 and n=91, respectively). Average methylation β-value for 'Low' methylation group patients = 0.19 (0.07-0.29) and for 'High' methylation group patients = 0.36 (0.30-0.59). **C-D**) Kaplan–Meier plots showing PFS (**C**) and OS (**D**) for the two patient clusters based on *FAM83A* expression using univariate Mantel-Cox log-rank survival analysis in HGSOC cohorts (Set 6, n=101 and n=102, respectively).

Figure S5

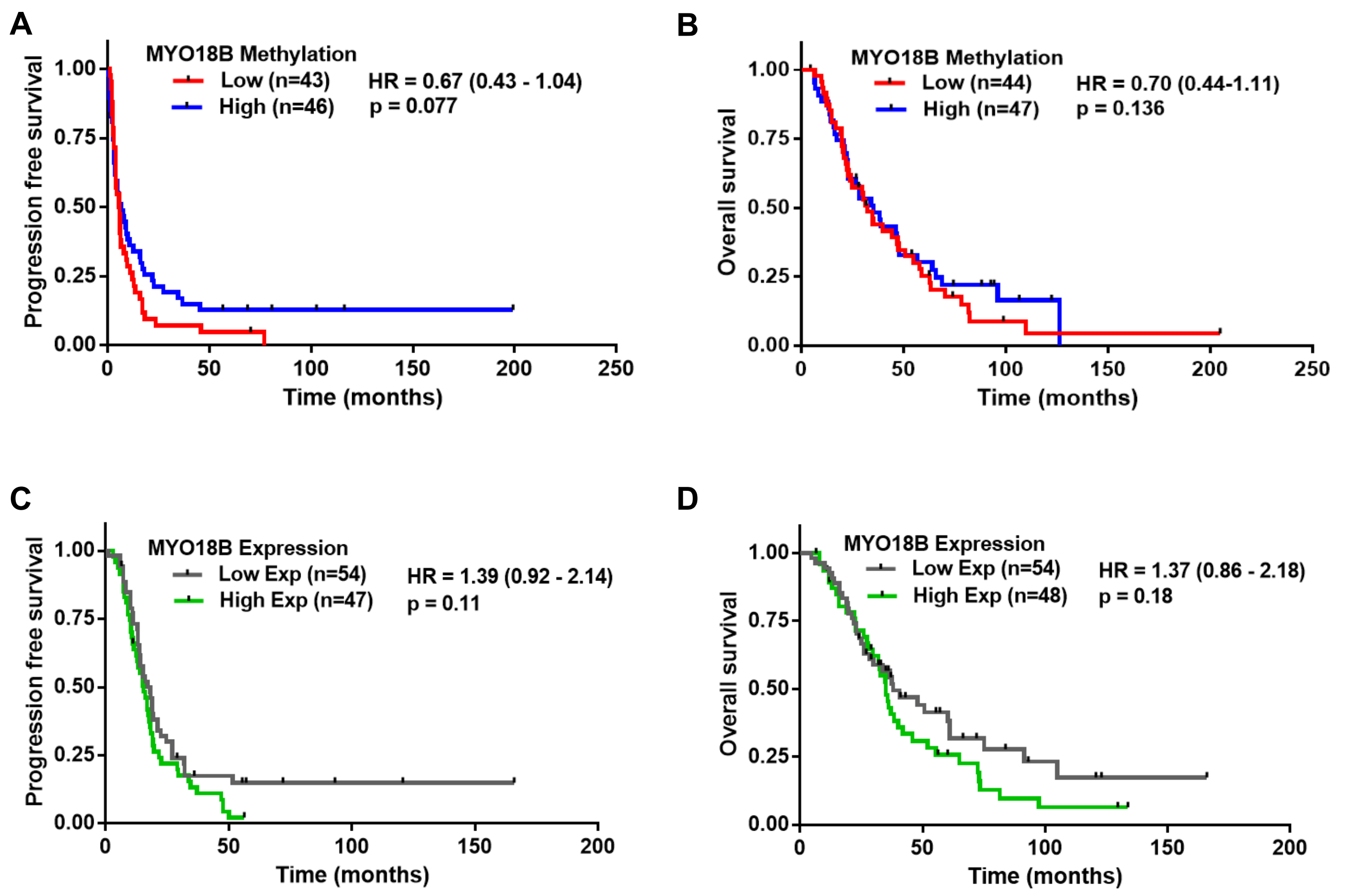

Figure S5. A-B) Kaplan–Meier plots showing PFS (**A**) and OS (**B**) for the two user-defined patient groups based on *MYO18B* methylation using univariate Mantel-Cox log-rank survival analysis in HGSOC cohorts (Set 4, n=89 and n=91, respectively). Average methylation β-value for 'Low' methylation group patients = 0.47 (0.25-0.57) and for 'High' methylation group patients = 0.66 (0.58-0.74). **C-D**) Kaplan–Meier plots showing PFS (**C**) and OS (**D**) for the two patient clusters based on *MYO18B* expression using univariate Mantel-Cox log-rank survival analysis in HGSOC cohorts (Set 6, n=101 and n=102, respectively).

Figure S6

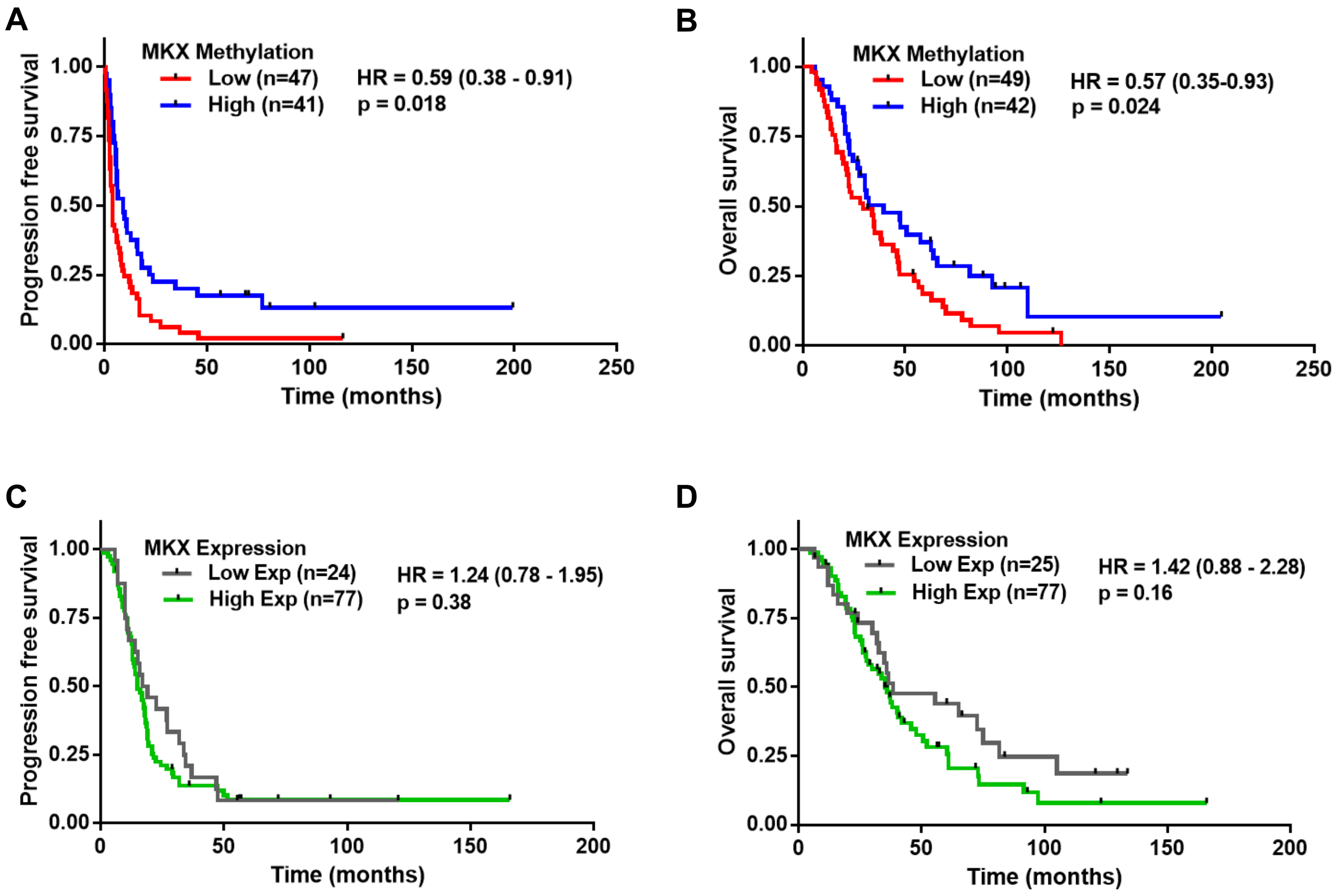

Figure S6. A-B) Kaplan–Meier plots showing PFS (**A**) and OS (**B**) for the two user-defined patient groups based on *MKX* methylation using univariate Mantel-Cox log-rank survival analysis in HGSOC cohorts (Set 4, n=89 and n=91, respectively). Average methylation β-value for 'Low' methylation group patients = 0.50 (0.17-0.65) and for 'High' methylation group patients = 0.74 (0.66-0.85). **C-D**) Kaplan–Meier plots showing PFS (**C**) and OS (**D**) for the two patient clusters based on *MKX* expression using univariate Mantel-Cox log-rank survival analysis in HGSOC cohorts (Set 6, n=101 and n=102, respectively).

Figure S7

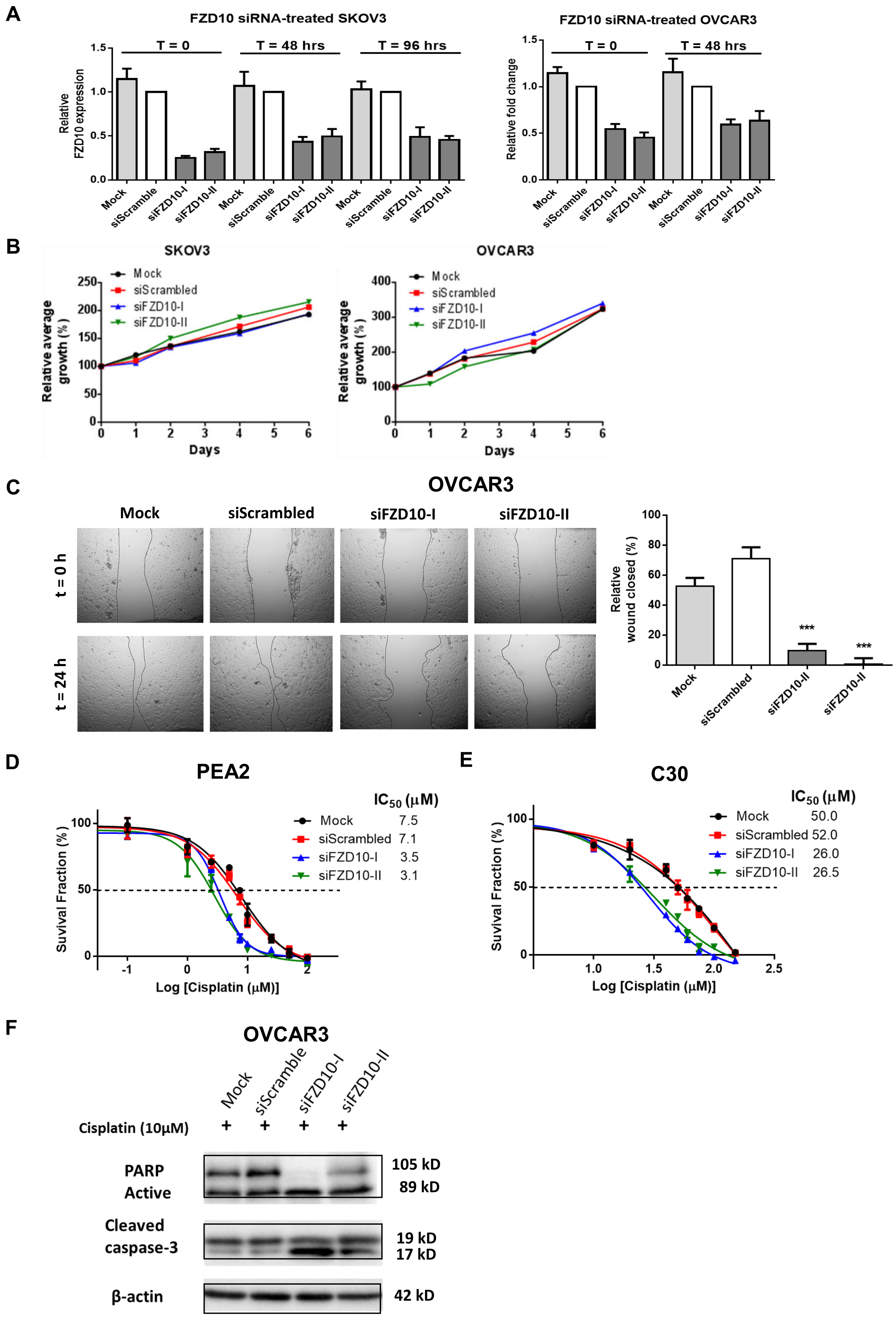
A
FZD10 siRNA-treated SKOV3
Relative FZD10 expression
T = 0
T = 48 hrs
T = 96 hrs
Mock
siScramble
siFZD10-I
siFZD10-II
FZD10 siRNA-treated OVCAR3
Relative fold change
B
SKOV3
OVCAR3
Relative average growth (%)
Mock
siScrambled
siFZD10-I
siFZD10-II
Days
C
OVCAR3
Mock
siScrambled
siFZD10-I
siFZD10-II
t = 0 h
t = 24 h
Relative wound closed (%)
***
D
PEA2
IC50 (μM)
Mock 7.5
siScrambled 7.1
siFZD10-I 3.5
siFZD10-II 3.1
Suvival Fraction (%)
Log [Cisplatin (μM)]
E
C30
IC50 (μM)
Mock 50.0
siScrambled 52.0
siFZD10-I 26.0
siFZD10-II 26.5
F
OVCAR3
Mock
siScramble
siFZD10-I
siFZD10-II
Cisplatin (10μM)
PARP
Active
105 kD
89 kD
Cleaved caspase-3
19 kD
17 kD
β-actin
42 kD

Figure S7. A) Relative mRNA expression of *FZD10* in SKOV3 and OVCAR3 cells treated with non-targeted scrambled siRNA (siScrambled) or two FZD10 targeted siRNAs (siFZD10-I and siFZD10-II), after 48 hrs of transfection (T=0), after 48 hrs of replating of siRNAs-treated cells (T=48 hrs) and after 96 hrs of replating (T=96 hrs). **B)** Growth curve of SKOV3 and OVCAR3 cells after treatment with siFZD10 in comparison to siScrambled or mock control over 6 days. **C)** Representative microphotographs (4x magnification) for wound healing assay on FZD10 siRNAs treated OVCAR3 cells for T=0 and T=24 hrs, along with the quantification of relative wound. Each bar represents % of wound closed ± SD from 3 independent experiments. *** $p<0.001$ for FZD10 siRNAs treated cells in comparison to the siScrambled, by student t-test. **D-E)** Short term MTT survival assay on siRNA treated PEA-2 **(D)** and C-30 cells **(E)** in the presence of cisplatin at indicated concentration after 96 hrs. * $p<0.05$; ** $p<0.01$ for siFZD10-I or siFZD10-II relative to scrambled control, Student t test. IC50 were calculated and are shown for each group in the inset. **F)** Protein levels of cleaved PARP and caspase 3 in OVCAR3 cells transiently transfected with either FZD10 siRNAs, along with treatment of cisplatin for 24 hrs for indicated concentrations.

Figure S8

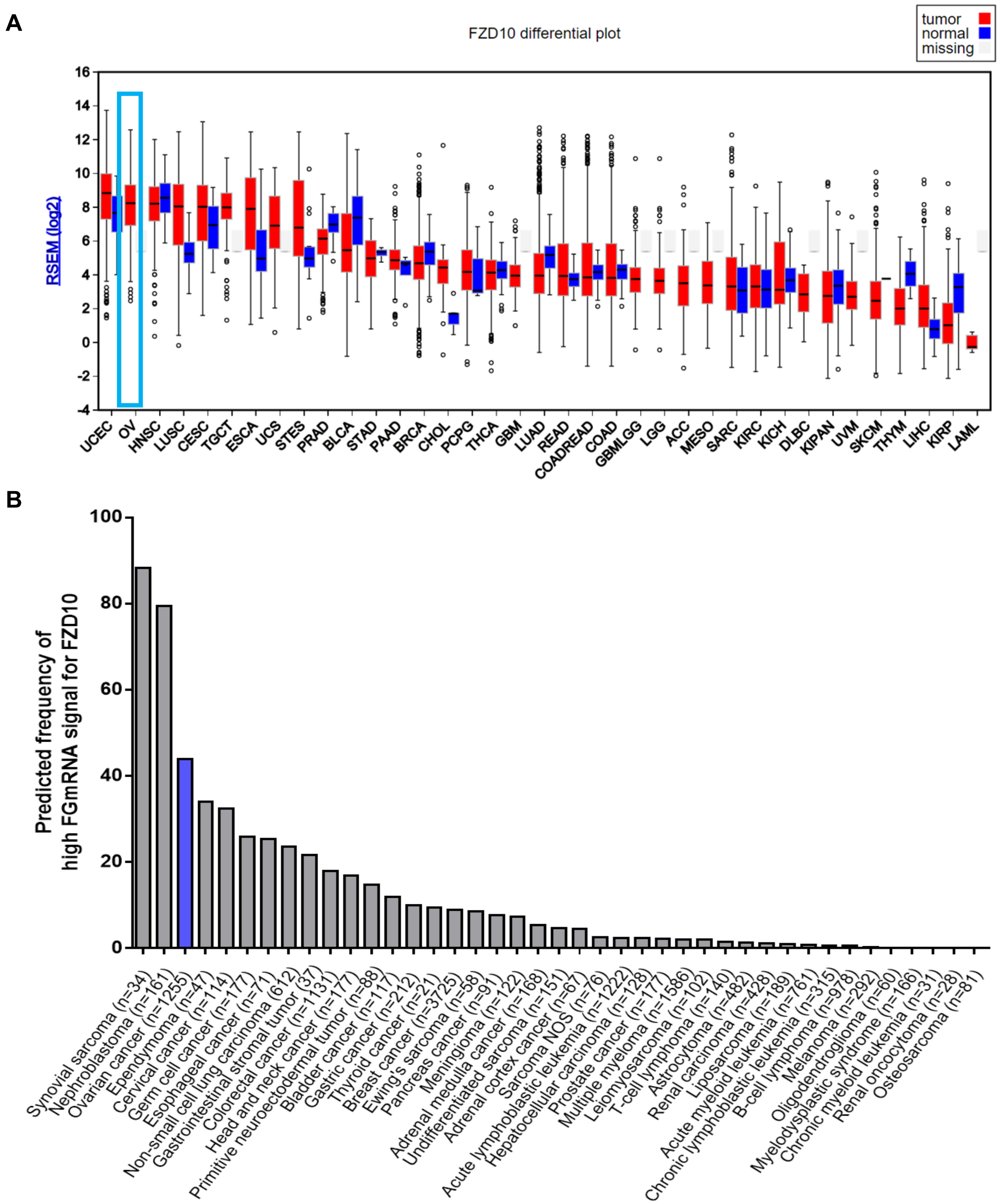

Figure S8. Global relative expression of *FZD10* **(A)** in different types of cancer based on the TCGA data*. **B)** *FZD10* predicted high expression level calculated with FGmRNA profiling [33] (see Material & Methods for details). The x-axis represents the percentage of samples per tumor type that show an overexpression of *FZD10*.

*Marx V. Drilling into big cancer-genome data. Nat Methods 2013;10:293–7
